# Demonstration of a 2–18 GHz Multispectral SAR

**DOI:** 10.3390/s26185737

**Published:** 2026-09-09

**Authors:** Mark A. Sletten, Jakov V. Toporkov, Steven P. Menk, Yanting Wang

**Affiliations:** U.S. Naval Research Laboratory, Remote Sensing Division, Washington, DC 20375, USA; mslettenmd@gmail.com (M.A.S.); steven.p.menk2.civ@us.navy.mil (S.P.M.); yanting.wang.civ@us.navy.mil (Y.W.)

**Keywords:** synthetic aperture radar, ultra-wideband radar, frequency-modulated continuous wave radar, sub-band processing, spectral signatures

## Abstract

This paper describes a polarimetric, frequency-modulated continuous wave (FMCW) synthetic aperture radar (SAR) with an ultrawide bandwidth that spans 2–18 GHz. It is being developed as an airborne sensor called SKuSAR. The intent is to generate a set of sub-band images, thereby creating a multispectral SAR. This opens the prospect for new remote sensing algorithms that exploit variations in the scene’s polarization/frequency response occurring over the system’s three octaves of bandwidth. We describe the SKuSAR hardware and the processing steps applied to the FMCW data to create a multispectral SAR. The approach is practically demonstrated using data collected against calibration targets deployed in a field with the system mounted on a truck. This ground-based arrangement provided an inexpensive solution to test and fine-tune the system hardware and processing algorithms. A few complicating factors specific to the ground-based geometry were encountered, such as multipath signal contamination due to ground reflection and rather short data collections that affected attainable azimuth resolutions. Both these factors were identified and analyzed. The measured characteristics follow theoretical predictions rather well, giving confidence that the system meets its expected nominal performance once airborne, with the mentioned limiting factors absent or of reduced significance.

## 1. Introduction

The growing availability of low-power, compact radars with extremely wide RF bandwidths has opened new possibilities for remote sensing. These ultra-wideband (UWB) systems use a frequency-modulated continuous wave (FMCW) waveform to achieve centimeter-scale range resolution. To date, these systems have been deployed primarily as altimeters to measure snow depth [1,2,3,4]. However, these radars are fully coherent and can be operated in a side-looking configuration to form a synthetic aperture radar (SAR) that can potentially support extremely fine spatial resolution. To reduce interference with other users of the RF spectrum, these UWB radars must have low average power, thus limiting their operating range/altitude. However, their small size and low weight would allow the accommodation of this restriction through the use of a high-performance commercial drone [5]. The resulting unmanned, day/night sensor system promises both civilian and military applications.

Historically, UWB SAR systems have been used for tasks that require both fine spatial resolution and penetration into a medium, with through-canopy sensing and buried mine detection being examples [6,7,8]. Fine spatial resolution requires high bandwidth while penetration requires lower frequencies, and thus the combination of the two necessitates a UWB system for which the bandwidth is comparable to the center frequency. But rather than high spatial resolution, our interest lies in developing applications that exploit variations in the scene response that may occur across the wide percentage bandwidth. For example, recent analysis indicates that a channelized, multispectral approach to the processing of UWB, along-track interferometric SAR data can improve the precision of ocean surface current estimates [9]. Recent research also indicates that variations in cross-track interferometric SAR coherence across a wide fractional bandwidth promise to provide measurements of the three-dimensional structure of semi-transparent media, such as a forest canopy [10,11]. A new system is also under development to exploit variations in the frequency response of snow to estimate depth, density, and snow water equivalent [12]. The multispectral analysis of UWB SAR data indicates that resonances may provide a new approach to the detection and characterization of man-made materials [13]. The growing availability of UWB radars is expected to stimulate interest in applications that exploit variations in the frequency response of the scene and of targets within it. For example, compact targets may display resonance modes at certain frequencies, while spectral signatures of surfaces would carry information on their roughness at different scales.

It is worth noting that many current satellite SAR systems, operating in spotlight mode, can provide very fine azimuth resolutions [14]. The latter, in turn, can be traded for a time-lapse image sequence through sub-aperture processing, e.g., [15,16]. This parallels the present idea of a UWB system trading the full-band range resolution for a less-sharp “image cube” that extends in the spectral dimension. In the optical domain, such an approach has been pursued by hyperspectral sensing, e.g., [17]. A multi-channel analysis across the wide frequency band has also been employed in microwave radiometry [18,19].

In this paper, we describe the UWB FMCW system we have developed to support this multispectral SAR research. The system spans the S- to Ku-bands from 2 to 18 GHz, and thus, we refer to it as SKuSAR. Because of the low-power constraints (to not interfere with other spectrum users), this is a “niche” sensor bound to operate at low altitudes/short distances (ideally, on a drone). The hardware is described in the next section, followed by a description of how the UWB FMCW data are channelized to form a multispectral SAR. In preparation for airborne deployment (which is the ultimate goal), a cost-effective solution to test the system performance and verify the processing approach using a moving truck was adopted. We describe the experimental setup and then present example imagery and an analysis of calibration target responses that demonstrate the system’s ability to measure the frequency and polarization response of objects within the scene. Expected and attained resolutions are also discussed. It is recognized that the truck-based operational conditions are rather different in many respects from what the airborne system would encounter. Those differences and the expectations for the aircraft-based sensor are discussed at the conclusion of the paper.

## 2. System Description

### 2.1. Hardware

**Figure 1:**
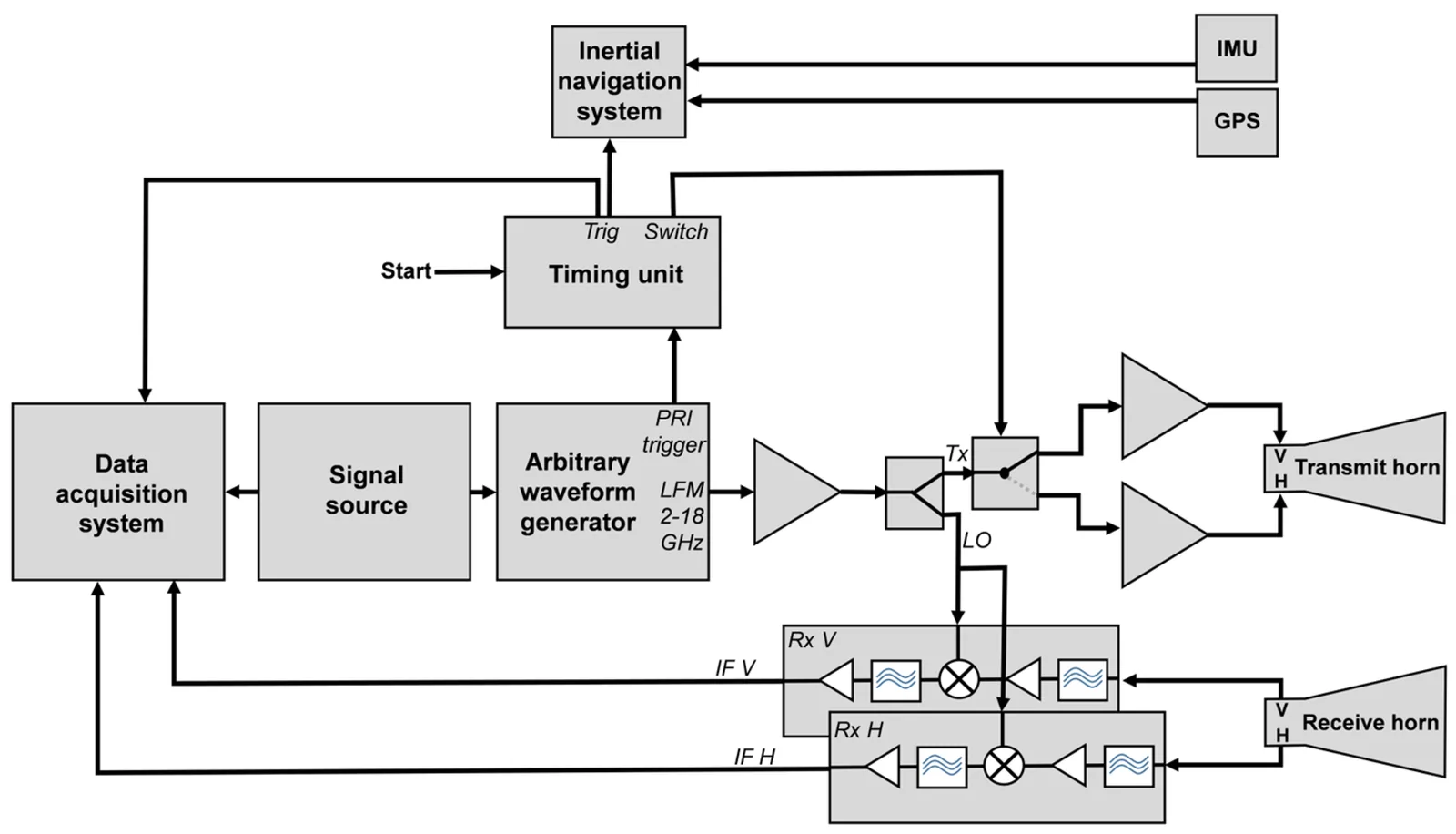
Block diagram of the SKuSAR system.

The block diagram of the system (Figure 1) follows the outline in [4] and is built around an arbitrary waveform generator (AWG). The latter generates a repetitive 2–18 GHz linear frequency-modulated (LFM) waveform that is amplified and split into the transmit (Tx) and local oscillator (LO) signals. For the data presented in this paper, both the pulse repetition interval (PRI) and the chirp duration were 248 μs, resulting in a chirp rate of 64.5 GHz/ms (note that while SKuSAR is a CW radar as opposed to a truly pulsed system, we occasionally use “pulse” to refer to a period of the repeated LFM waveform). The Tx signal is routed through a switch to generate two interleaved transmit channels that are fed through final power amplifiers to the vertically (V) and horizontally (H) polarized ports of an ultra-wideband, quad-ridged horn antenna. The average power for each polarization channel is approximately 1 watt. A separate dual-polarized horn is used as a receive antenna. The V and H receive signals are fed into separate receive modules (Rx V and Rx H), where they are mixed with the LO to generate intermediate frequency (IF) signals. The receive modules include a bandpass IF filter, which produces a range swath of approximately 140 m (nominally covering ranges between 440 m and 580 m, although the drop-off on each side is not abrupt)—see, e.g., [20] (Chapter 3) and Equation (1). The sampling rate $f_{s}$ at which the IF signals are digitized and recorded affects the available unaliased range (cf. Appendix A) and should be chosen in concert with the above-mentioned swath-defining filter. The data acquisition system (DAS) and the AWG are fed by the synchronized clocks of the signal generator. The timing unit uses a PRI trigger along with a manual start signal to produce a trigger for DAS, the control signal for the polarization switch, and a marker for the inertial navigation system (INS). The latter blends the data from the GPS and the inertial measurement unit (IMU) to supply precise measurements of the antenna positions as the SKuSAR platform moves.

**Figure 2:**
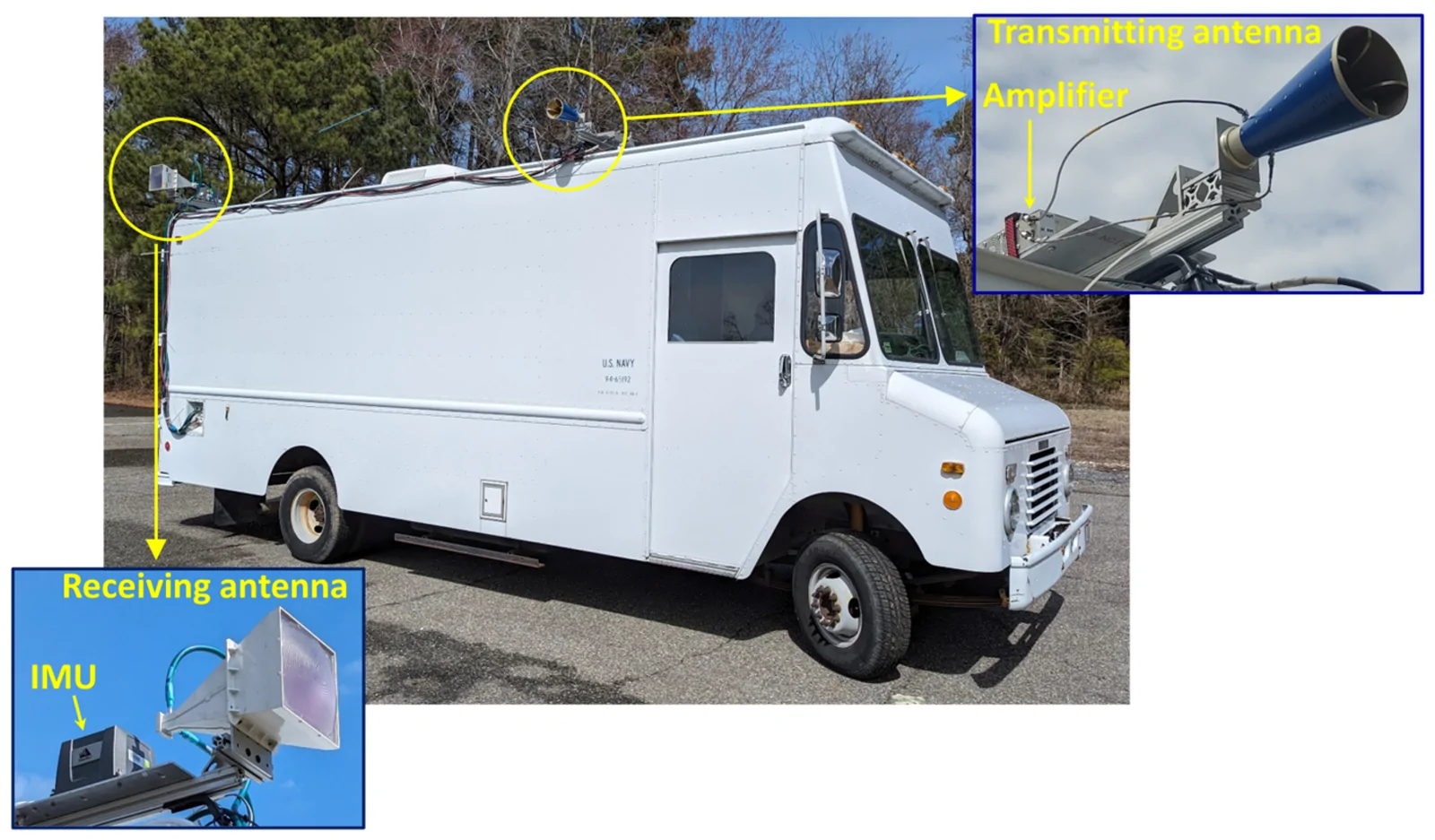
Photograph of the truck used as a SKuSAR platform, highlighting the positioning of the system antennas and IMU.

The eventual goal is to integrate SKuSAR onto a UV-18 aircraft, the Navy version of a commercial Twin Otter, and fly it. To gain a low-risk, inexpensive workbench to test both the assembled system and the developed processing tools, the radar was installed and operated on a truck, as shown in Figure 2. All data presented in this paper were collected using this vehicle as a moving platform. The transmit and receive antennas are mounted on the roof of the truck with a spacing of 4.8 m, a distance that is sufficient to reduce direct coupling between the antennas to a negligible level. The final power amplifier is mounted next to the transmit antenna, while the GPS antenna and IMU are placed directly behind the receive horn, near the rear of the vehicle. All other electronics are located inside the truck, and the entire system is powered by a built-in diesel generator.

### 2.2. Data Processing

**Figure 3:**
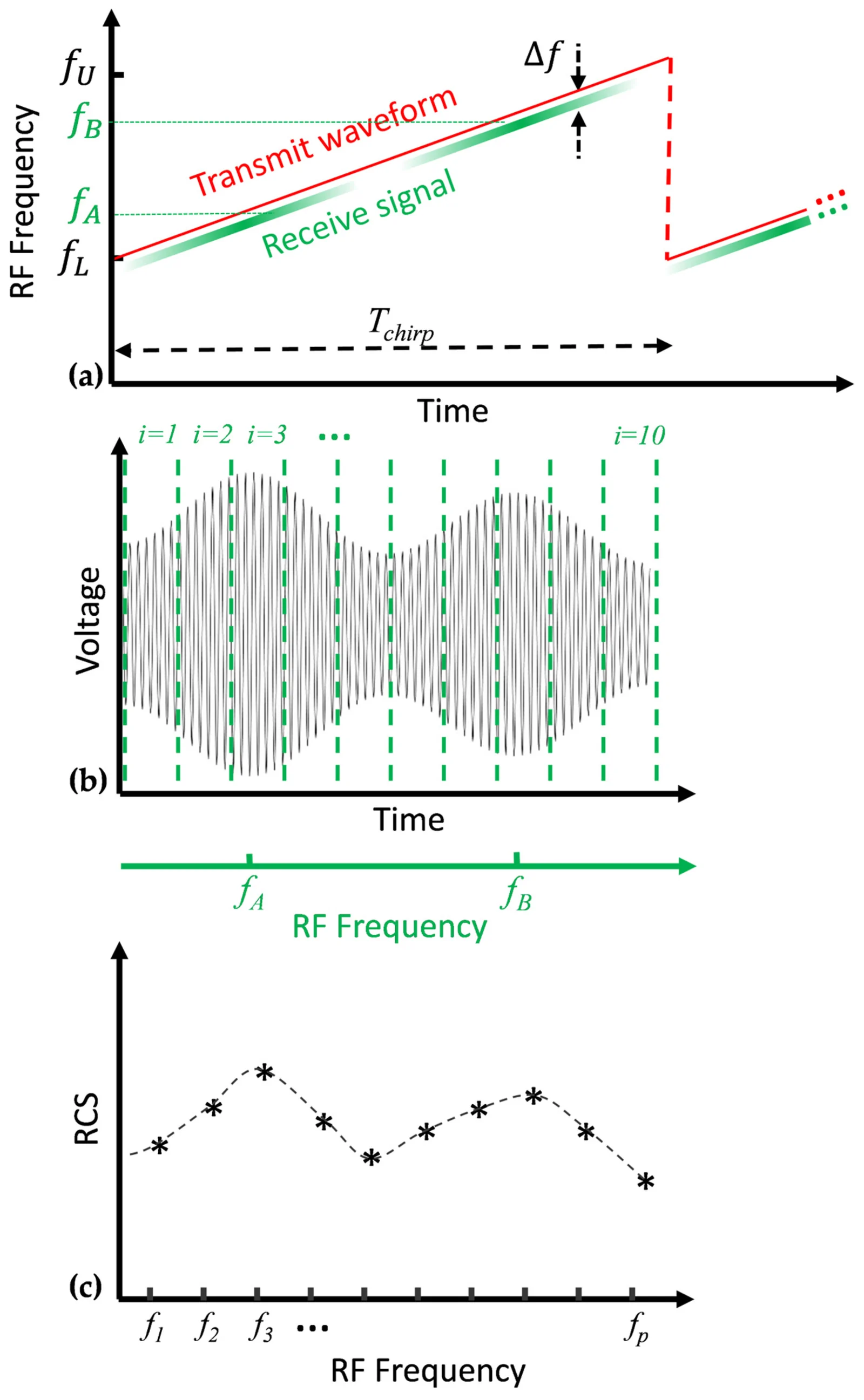
Illustration of sub-band treatment of UWB FMCW data for a single target whose frequency response has peaks at $f_{A}$ and $f_{B}$ (see [9]). (**a**) Transmitted (red) and received (green) waveforms vs time; (**b**) IF signal with assigned RF sub-bands; (**c**) derived RF frequency response.

Figure 3 illustrates how UWB FMCW radar data can be conveniently partitioned into RF sub-bands (also cf. [9]). For simplicity and purposes of illustration, the scene is assumed to contain a single point target that exhibits two peaks in its RF frequency response, located at frequencies $f_{A}$ and $f_{B}$.

As shown in Figure 3a, the radar system transmits a repetitive LFM chirp (red trace) of duration $T_{chirp}$ that begins at frequency $f_{L}$ and ends at $f_{U}$. The received signal (green trace) is a delayed version of the transmitted chirp with an amplitude that is modulated in accordance with the frequency response of the target. The intensity of the green trace in Figure 3a indicates this modulation. The round-trip propagation delay between the radar and the target introduces a constant-frequency shift, Δf, between the transmitted and received signals at any point in time. This shift is proportional to the radial distance between the radar and the target,

(1) R=cΔf/2α

where

(2) $$\alpha ={\left(f_{U}-f_{L}\right)/T_{chirp}=B}_{tot}/T_{chirp}$$

is the LFM chirp rate, $B_{tot}=f_{U}-f_{L}$ is the total chirp bandwidth, and c is the speed of light, e.g., see [20] (Chapter 3). For the SKuSAR data presented in this paper, $f_{L}=$ 2 GHz, $f_{U}=$ 18 GHz, and $T_{chirp}=PRI=$ 248 μs.

In the FMCW radar receiver, the received signal is mixed with the LO (a copy of the transmitted waveform) and then low-pass filtered to form the IF signal, represented by the black trace in Figure 3b. The IF oscillates at the difference between the LO and receive frequencies. For a single point target, the IF signal in this illustration contains only a single frequency component at Δf (proportional to the target range, cf. Equation (1)). As shown in Figure 3b, the IF signal’s amplitude will be modulated according to the RF frequency response of the target (for the LFM waveform, time doubles as the RF frequency axis, as shown). RF sub-bands can be formed by simply dividing the data collected during each period of the IF into d segments, as indicated by the green vertical lines in Figure 3b. Ten sub-bands are assumed in this illustration (d=10), but any number of segments can be formed. Since a target at a given range results in a specific constant-frequency oscillation depicted in Figure 3b, the return from a collection of targets will be analyzed via fast Fourier transform (FFT) to distinguish contributions from different distances. The result (picked in this manner at the range of our single target) is shown conceptually in Figure 3c, where the maxima in the modulation at $f_{A}$ and $f_{B}$ observed in Figure 3b result in the peaks in the radar cross section (RCS) in the corresponding sub-bands. The mathematical treatment of the FMCW range compression procedure is provided in Appendix A.

If the bandwidth of each sub-band is set to $B_{tot}/d$, this translates into a coarser (by the factor of *d*) range resolution, as reflected in (A8) and (A12). The signal duration $T_{sub}$ also becomes shorter, cf. (A11). The signal-to-noise ratio (SNR) upon range compression is proportional to $T_{sub}$ (which is essentially the coherent integration time), indicating SNR reduction by the factor of d. Thus, the multispectral capability comes at the expense of sensitivity and achievable range resolution. In our case, the 1-GHz sub-bands appeared as a good compromise that allowed a relatively detailed examination of the backscatter frequency dependence while resulting in acceptable signal-to-noise properties for the rather bright targets. The expected 15 cm range resolution would also yield a number of independent samples to suppress speckle for extended targets and scenes with a larger sliding window. The issues behind selecting and combining sub-bands were quantitatively considered in [9] with a focus on along-track interferometric SAR applications. The sub-bands can be selected with an overlap to provide for denser spectrogram samplings—here, a 0.5-GHz stride is generally used. Using finer strides essentially implements the sliding spectral window but would entail higher computational costs.

Actual scenes contain many scattering centers located at many different distances from the radar, and thus, as mentioned, the IF signal will constitute a coherent mixture of modulated sinusoids at many different frequencies. Segmenting the IF signal as indicated, followed by FFTs on each individual sub-band, will create the coherent range profile of the backscatter for each RF sub-band. To reduce sidelobes, a Hamming window is applied on each IF signal segment, essentially replacing the rectangular windows in (A6) and (A9). As implemented, the size of the FFT is kept the same (198,400 points, with zero-padding for the samples outside the sub-band window)—this optional step produces oversampled outputs on the same range grid. For each pulse (LFM period), the procedure will form a two-dimensional array of data in range and RF frequency (specifically, sub-band central frequencies). With the radar mounted on a moving platform along with instrumentation to measure its motion, these range profiles can then be fed into a SAR processor to form a set of SAR images, one for each sub-band (some consequential phase factors, though, do emerge and need to be compensated, cf. Appendix A). Any algorithm that has the range compression operation upfront should fit. With SKuSAR, we re-purposed a time-domain back-projection (BP) processor for image formation (e.g., [21]), implemented in Matlab (v. R2024b) with parallel processing functions to speed up computations.

## 3. Example Data

### 3.1. Example Image

**Figure 4:**
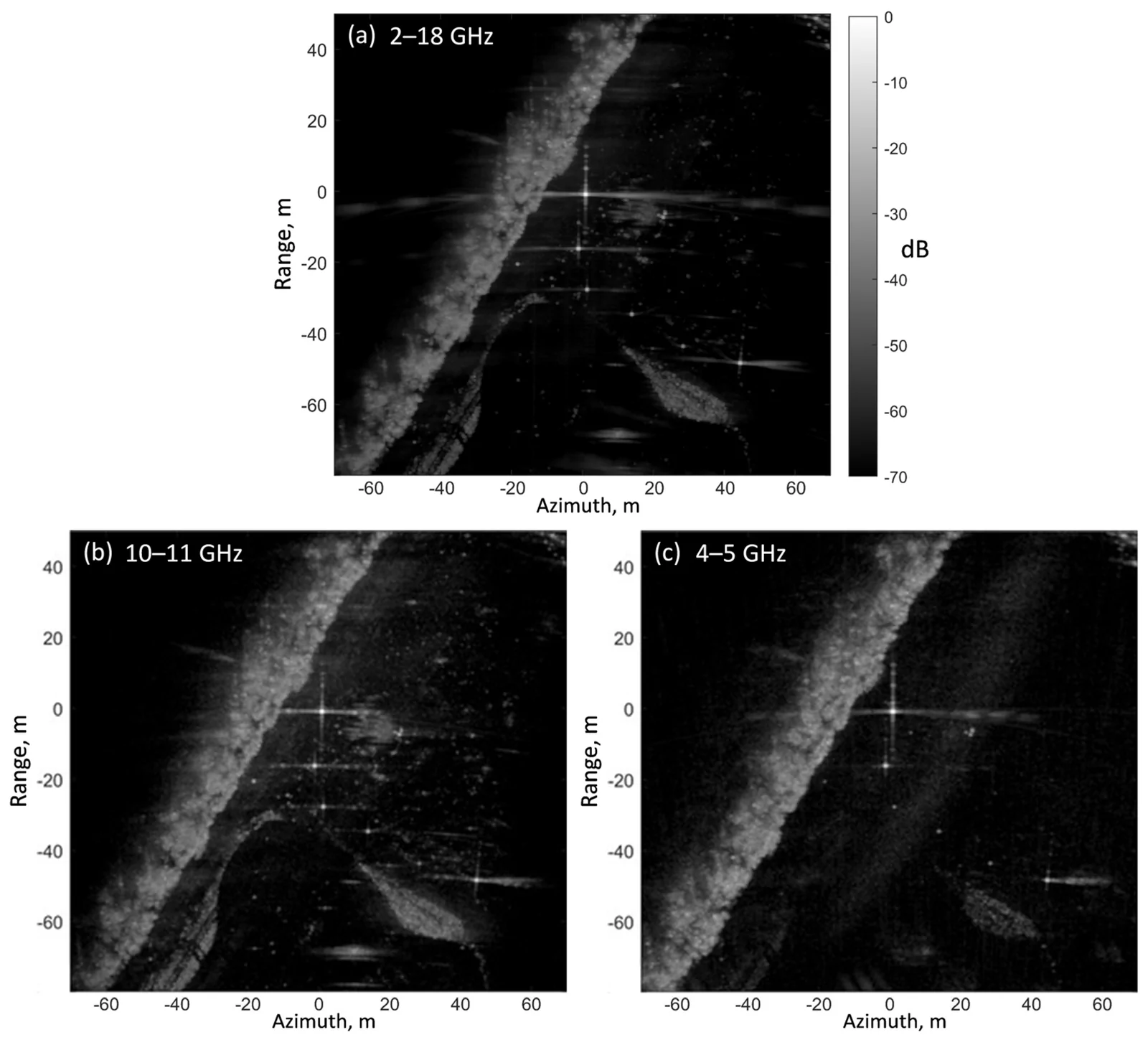
Sample SKuSAR images (VV polarization) of a test site in Southern Maryland, smoothed to 0.5 m spatial resolution. The diagonal band is a tree line, while the bright features near the center of the image are produced by corner reflectors. (**a**) Full-band image; (**b**) 10–11 GHz sub-band; and (**c**) 4–5 GHz sub-band.

**Figure 5:**
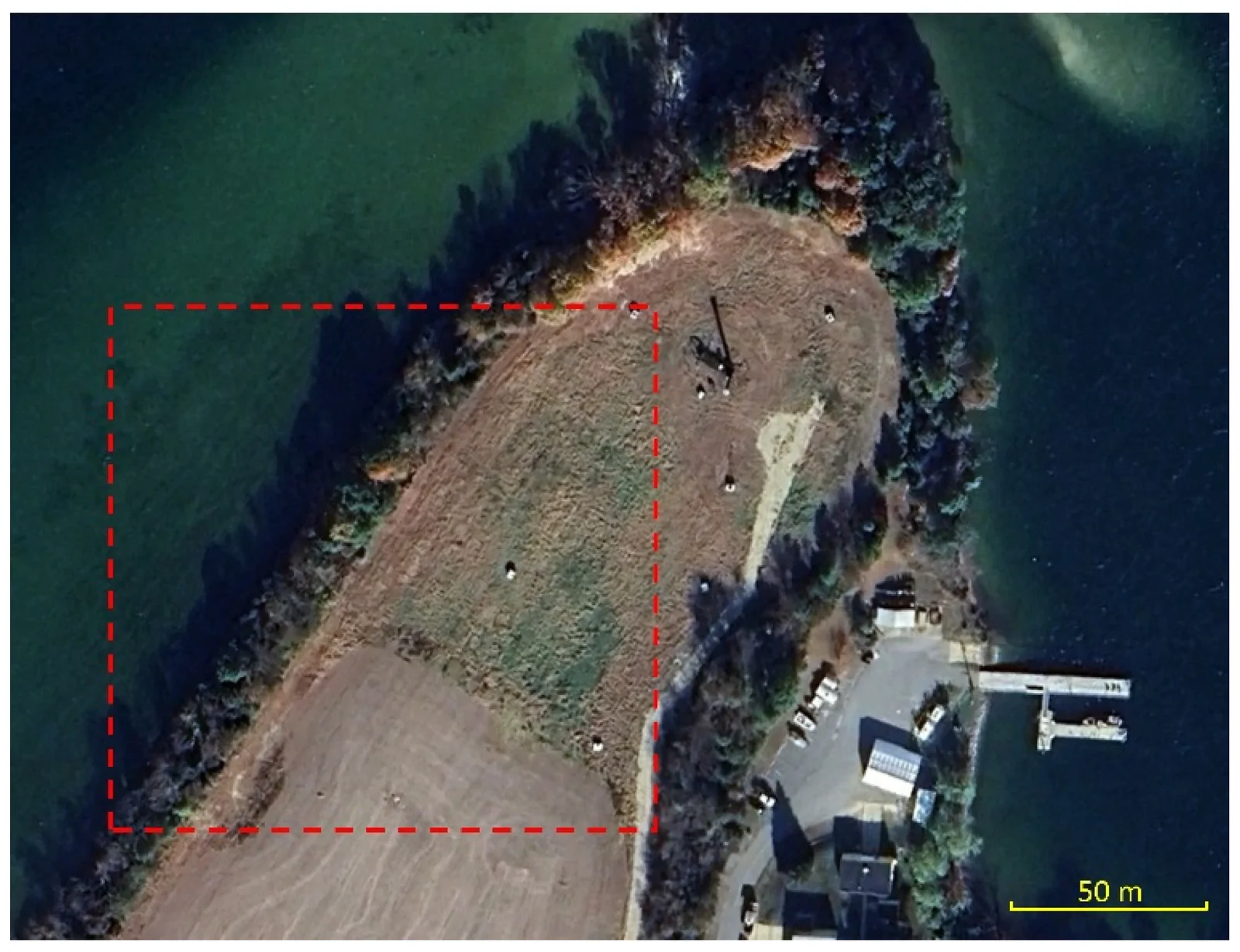
Google Earth image of the test site in Southern Maryland. The area within the image in Figure 4 is outlined in red.

Figure 4 contains sample VV SKuSAR images, generated from data collected against a number of calibration targets deployed in a field in Southern Maryland. They were processed using full system bandwidth (Figure 4a) as well as with the sub-bands of 10–11 GHz (Figure 4b) and 4–5 GHz (Figure 4c). A Google Earth view of the area is shown in Figure 5, with the image boundary outlined by the red box. In addition to the tree line, the curved boundary of the bare field can be seen in the lower half of the images and several bright calibration targets are prominent in the central area. The topography of the scene is essentially flat. The images were smoothed to a spatial resolution of 0.5 m, while the data were collected over a linear aperture length of 67 m with an average vehicle speed of 8.4 m/s. The range to the images’ coordinate origin is 480 m.

**Figure 6:**
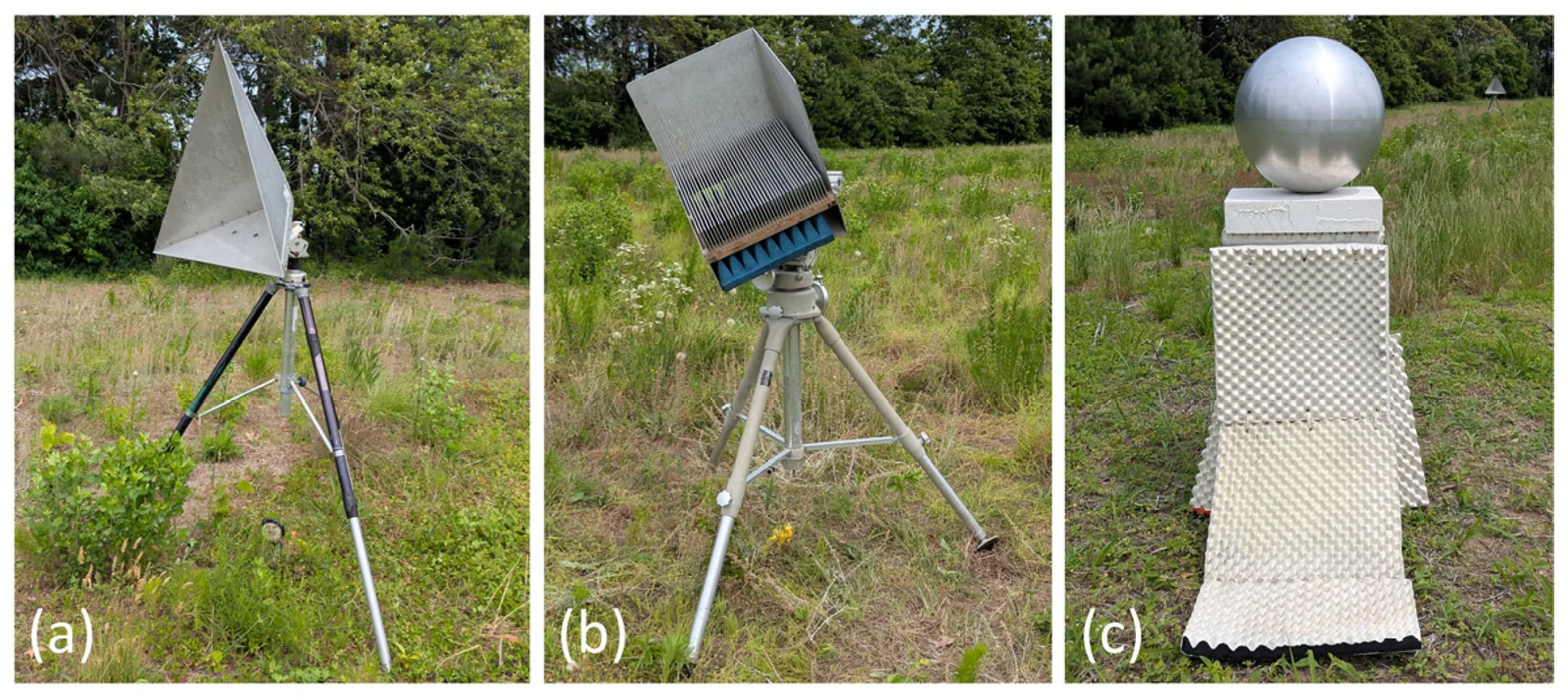
SKuSAR calibration targets: (**a**) 24″ standard trihedral corner reflector; (**b**) 15″ gridded trihedral corner reflector; and (**c**) 20″ diameter sphere.

The calibration targets used to evaluate SKuSAR system characteristics are shown in Figure 6. These include a 24-inch standard trihedral corner reflector (Figure 6a), a 15-inch gridded trihedral (Figure 6b), and a 20-inch diameter calibration sphere (Figure 6c). Given the relatively low cross section of the sphere, the tripod used to support it was covered with the white radar absorber visible in the photo. The standard trihedral and the sphere are common radar calibration targets that (ideally) produce only co-polarized backscatter, that is, backscatter with the same polarization as the incident radiation. The observed cross-polarization levels serve as an indicator of the expected polarization isolation. In contrast, the gridded trihedral is designed to generate both co- and cross-polarized backscatter, with the latter referring to backscatter that is orthogonally polarized with respect to the incident radiation. The depolarizing response is produced by replacing one wall of a standard trihedral with a parallel plate grid on top of a layer of radar absorber, both of which are visible in Figure 6b. This is a variation in the original design introduced by Sheen et al. [22].

### 3.2. Corner Reflector Response

**Figure 7:**
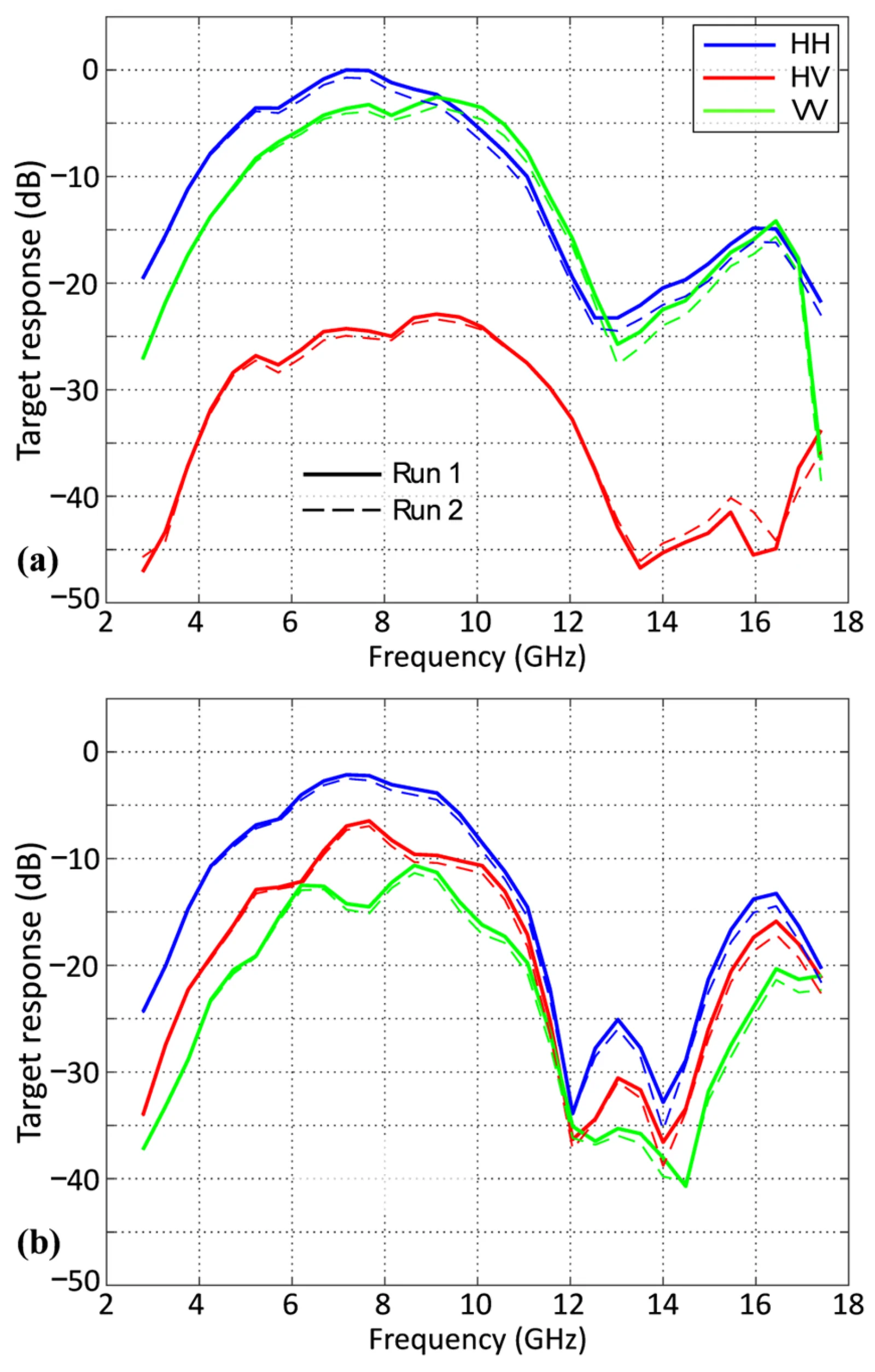
Trihedral corner reflector responses measured during two consecutive runs: (**a**) standard corner reflector and (**b**) gridded corner reflector. All curves are normalized to the maximum HH response in panel (**a**).

The observed responses of the standard and gridded trihedrals are shown in Figure 7 for both horizontal (HH) and vertical (VV) co-polarization as well as cross-polarization (HV). The curves in both panels are normalized to the common highest value that occurs in the HH response of the standard trihedral. To produce these results, for each polarization combination, data covering the full 2–18 GHz bandwidth were first partitioned into segments of 1 GHz with a 0.5-GHz overlap in the manner described above in Section 2.2. An oversampled (at 2 cm pixel spacing) SAR image corresponding to each segment was produced, and the peak value of the target response was located and measured. The figure includes the measurements obtained during two consecutive runs, demonstrating good consistency.

While contaminated with system polarization distortions, the measured signatures are nominally in agreement with those expected from theory. As shown in Figure 7a, the VV and HH responses of the standard trihedral are roughly equal while their cross-polarized counterpart is approximately 20–25 dB lower. The difference between VV and HH curves indicates the system imbalance associated with transmit power, antenna gain, and transmission loss. The non-vanishing cross-polarization response signifies the presence of crosstalk coupling between two orthogonal polarization states. The incurred polarization impurity, however, has a negligible impact on the co-polarization responses that are studied hereinafter. In contrast, the gridded trihedral produces a strong response in all polarizations, as shown in Figure 7b, with the cross-polarization return exceeding the VV one (and predicted to be 4.8 dB higher in theory for the chosen configuration).

**Figure 8:**
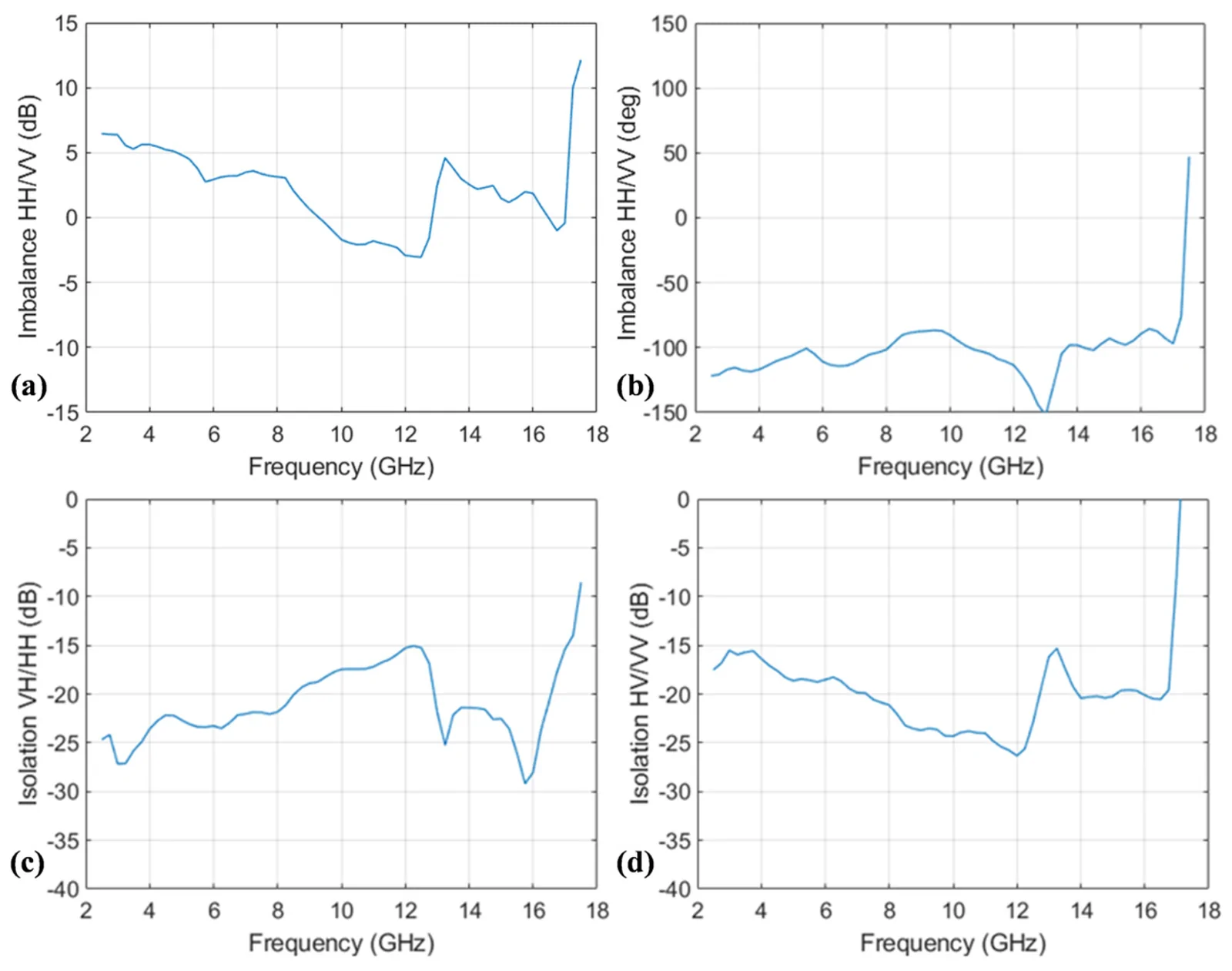
Spectral variation in system polarimetric merits estimated from the trihedral pixel in the truck-collected SAR images: inter-polarization imbalance between HH and VV in both magnitude (**a**) and phase (**b**), and cross-polarization isolation at H-pol transmission (**c**) and V-pol transmission (**d**). The source images were formed using 1 GHz sub-bands with a 0.25 GHz stride.

We note significant variations with frequency both in terms of the intensity for each polarization state and in terms of the relative changes between different polarizations. The former effect is not only attributed to changes in transmitted power and system gains but also greatly to the multipath interference (to be discussed in the next subsection). The latter demonstrates a challenge particular to an ultra-wideband polarimetric system: the polarization distortions migrate with frequency, as highlighted in Figure 8 based on the measured standard trihedral response. Both the co-polarization imbalance and cross-polarization isolation levels (defined as ratios of signals in the corresponding channels) vary across the available bandwidth: up to 8 dB (from –3 dB to +5.6 dB) in imbalance and 10 dB (from –25 dB to –15 dB) in isolation. In this regard, sub-band processing provides a viable solution to calibrate the polarimetric observations for intrinsic target signatures. The available techniques include those using standard calibration targets [23] and ones relying on clutter and reflection symmetry [24]. However, considering the restricted imaging geometry during the truck-based collection (more on that in Section 4), in this paper, we focus on the evaluation of the SKuSAR system response and defer polarimetric calibration to the later airborne collection where we can maintain consistent dwell across the whole scene.

### 3.3. Multipath Analysis of Sphere Response

The responses of both trihedrals shown in Figure 7 exhibit a prominent notch in excess of 20 dB between 12 GHz and 14 GHz. In this section, we analyze similar notches in the response of the sphere to show that they are, in fact, multipath nulls with locations that depend on the height of the sphere above ground.

The measured response of a target in the scene, $S_{meas}\left(f\right)$, as a function of RF frequency is

(3) $$S_{meas}\left(f\right)=T_{system}\left(f\right)\cdot S\left(f\right)$$

where $T_{system}\left(f\right)$ is the frequency-dependent transfer function resulting from the SKuSAR hardware and data processing, and $S\left(f\right)$ is the true response of the target. For the specific case of the sphere on a tripod, we model $S\left(f\right)$ as the product of the scattering coefficient of an isolated sphere, $S_{0}^{sphere}\left(f\right)$, and a multipath factor, $T_{mp}\left(f,h_{s}\right)$, that depends on both frequency and the height $h_{S}$ of the sphere above the (assumed to be flat) ground:

(4) $$S\left(f\right)=S^{sphere}\left(f,h_{s}\right)=T_{mp}\left(f,h_{s}\right)\cdot S_{0}^{sphere}\left(f\right).$$

Over the SKuSAR band, the theoretical response of the 20-inch sphere in isolation is essentially a constant, $S_{0}^{sphere}\left(f\right)=A$. Assuming that the multipath factor is known or can be accurately modeled (see below), the SKuSAR system transfer function can then be determined from

(5) $$T_{system}\left(f\right)=\frac{S_{meas}\left(f\right)}{S\left(f\right)}=\frac{S_{meas}^{sphere}\left(f,h_{s}\right)}{T_{mp}\left(f,h_{s}\right)\cdot A}.$$

**Figure 9:**
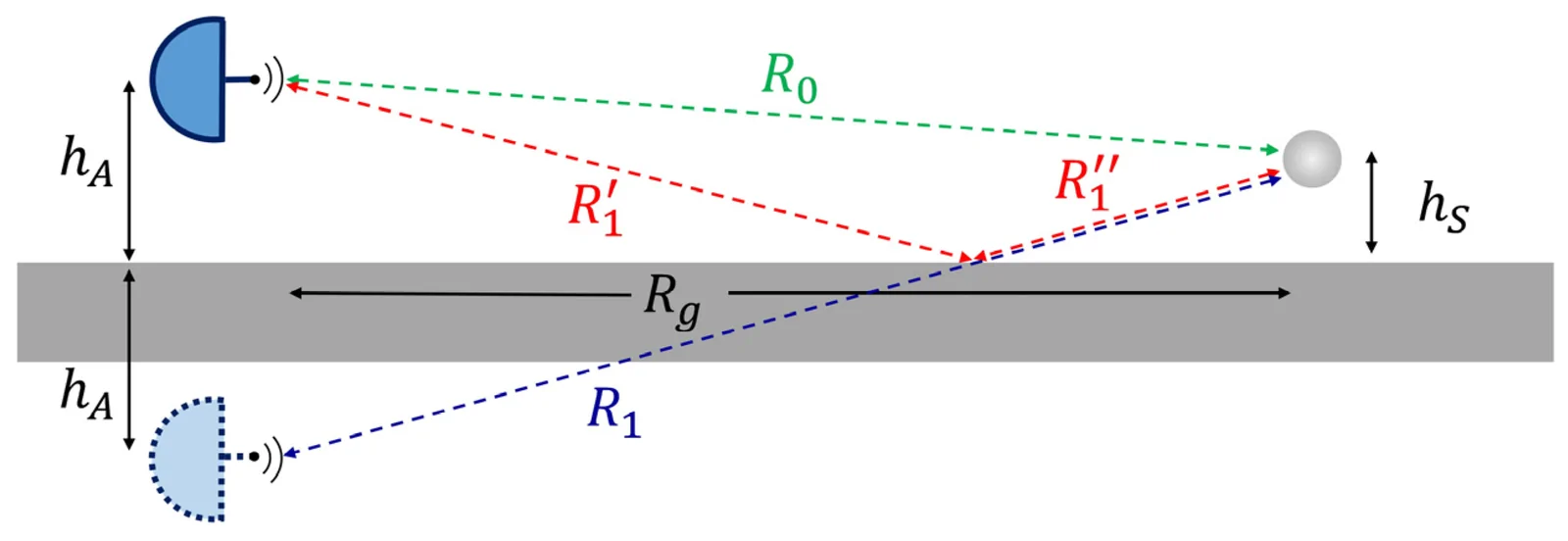
Sketch defining parameters used in the multipath model.

Using simple geometry, a ray-optics approach and the image theory, the received signal produced by the sphere in the presence of multipath can be modeled as (e.g., see [25])

(6) $$S^{sphere}=A\left[exp\left(\frac{i4\pi fR_{0}}{c}\right)+{2\Gamma }_{g}exp\left(\frac{i2\pi f\left(R_{0}+R_{1}\right)}{c}\right)\left.+{\Gamma_{g}}^{2}exp\left(\frac{i4\pi fR_{1}}{c}\right)\right]=AT_{mp},\right.$$

where

(7) $$R_{0}=\sqrt{{\left(h_{A}-h_{S}\right)}^{2}+R_{g}^{2}}$$

(8) $$R_{1}\equiv R_{1}^{\prime }+R_{1}^{”}=\sqrt{{\left(h_{A}+h_{S}\right)}^{2}+R_{g}^{2}}$$

and $T_{mp}$ is the multipath factor. As illustrated in Figure 9, $h_{A}$ is the height of the radar antenna above the ground level and $R_{g}$ is the distance along the ground plane between the radar and the target. $\Gamma_{g}$ is the forward reflection coefficient of the ground (generally a complex quantity) whose value depends on soil permittivity, surface roughness, vegetation, and grazing angle. For a low-grazing configuration, it becomes real-valued and negative [26]. The first term in (6) represents the signal component that travels directly between the radar and the sphere. The second and third terms represent components that follow ray paths that include a “bounce” off the ground during either the outbound or return segment of the round-trip signal path (second term) or a ground bounce during both the outbound and return segments (third term). Interference between these components causes peaks in the target response at frequencies for which the interference is constructive and notches where the interference is destructive. Note that for the extremely low-grazing angle configuration of our truck-based measurements, the multipath reflections that occur at the sphere involve very small bistatic angles, and thus, we assume that the corresponding bistatic scattering coefficients of the sphere are essentially equal to the monostatic coefficient, A.

The expression in (6) can be further rewritten to yield

(9) $${\left|T_{mp}\right|}^{2}={\left[1+{\left|\Gamma_{g}\right|}^{2}-2\left|\Gamma_{g}\right|\cos\left(\frac{2\pi f(R_{1}-R_{0})}{c}\right)\right]}^{2}≅{\left[1+{\left|\Gamma_{g}\right|}^{2}-2\left|\Gamma_{g}\right|\cos\left(\frac{4\pi fh_{A}h_{s}}{cR_{g}}\right)\right]}^{2},$$

where Taylor expansions of (7) and (8) were used to approximate the difference, $R_{1}-R_{0}$. Equation (9) elucidates the behavior of the multipath factor: it has maxima at

(10) $$f_{max}=\frac{(2n+1)cR_{g}}{4h_{A}h_{s}},n=0,1,2\ldots$$

and minima at

(11) $$f_{min}=\frac{ncR_{g}}{2h_{A}h_{s}},n=0,1,2\ldots$$

**Figure 10:**
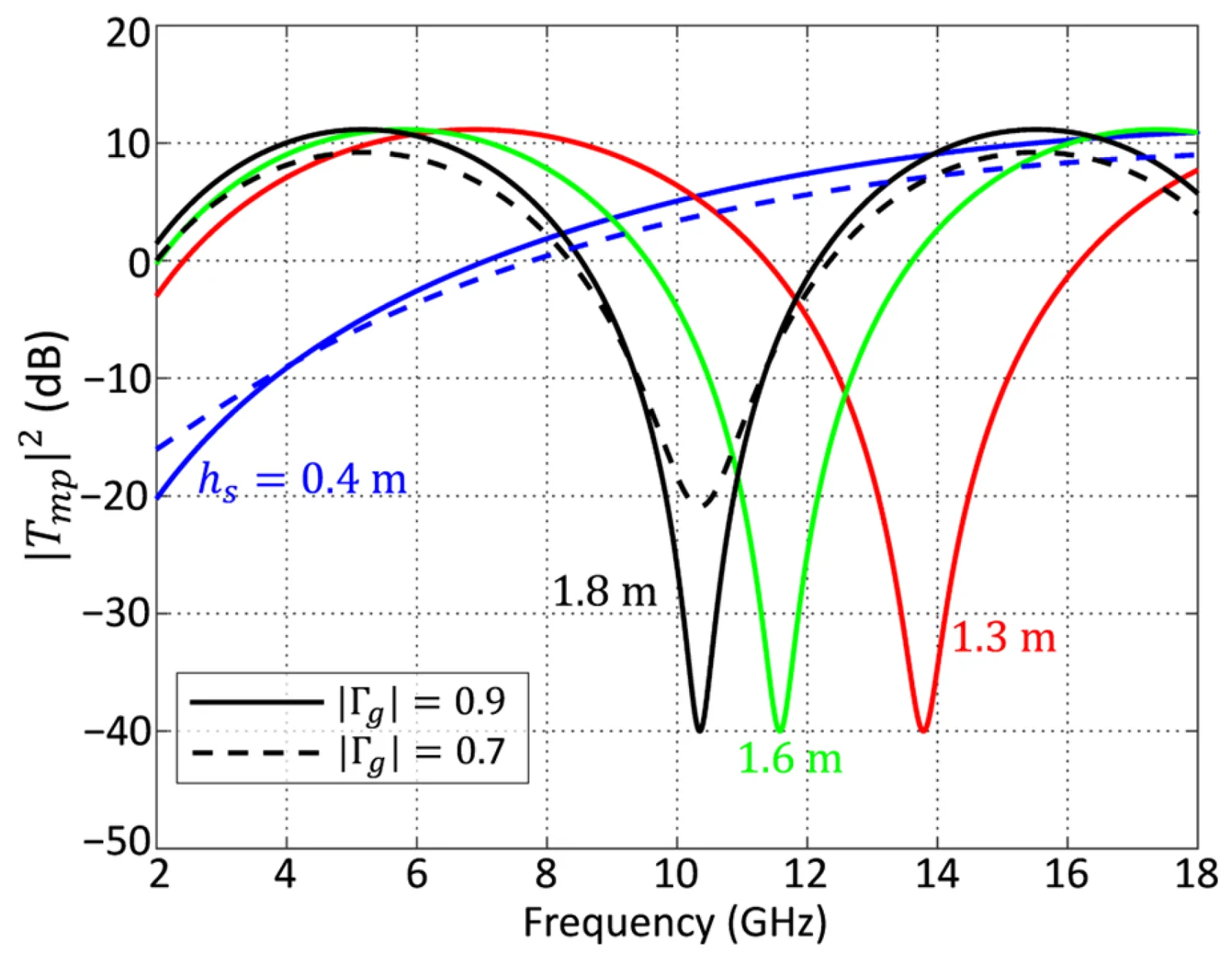
Theoretical multipath interference factor in Equation (9) for the sphere height positions used in the experiment. Solid lines correspond to the ground reflection coefficient magnitude of 0.9; for two height cases of 0.4 m and 1.8 m, calculations are also performed with $\left|\Gamma_{g}\right|=0.7.$

Those locations do not depend on $\Gamma_{g}$. For our frequency range, the pattern is shown in Figure 10, considering the sphere heights set during the experiment. In all calculations, we will use the measured values $h_{s}=$ 0.46 m, 1.33 m, 1.59 m, and 1.77 m, while referring to a “0.4 m sphere” or a “1.6 m height” in the text for brevity and convenience (the sphere height was changed between truck passes by adjusting the tripod covered by the radar absorber.) The plots use the ground broadside GPS-measured distance to the sphere, $R_{g}=460$ m, and the antenna height above the target plane, $h_{A}=3.75$ m. Notably, for the higher-positioned targets, we do get both the first maximum (n=0 in Equation (10)) and the first non-trivial minimum (n=1 in Equation (11)) within our frequency band. For $h_{s}=$ 1.8 m, the second maximum is observed at 15.3 GHz. In contrast, no maxima or minima fall within the system frequency range when the target is at the height of 0.4 m.

**Figure 11:**
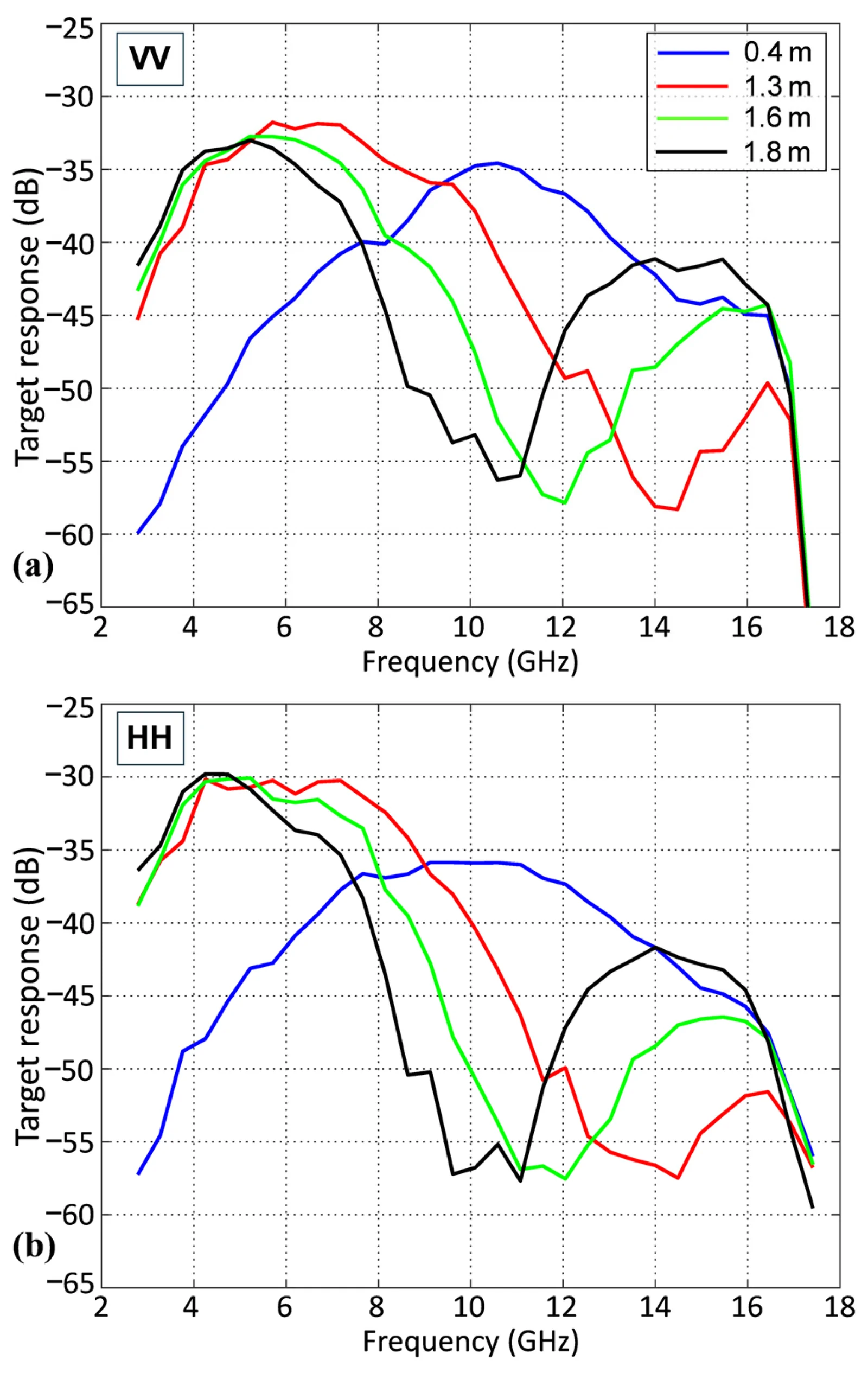
(**a**) VV and (**b**) HH sphere response when positioned at different heights above the ground. The plots in both panels are normalized to the maximum value of the data in Figure 7a.

The measured spectral response from the sphere is shown in Figure 11, with the sub-band width and the stride again being 1 GHz and 0.5 GHz, respectively. The curves in both panels are normalized to the maximum data value occurring in Figure 7a, highlighting a weaker sphere response compared to that of the corner reflector. Both HH and VV polarizations look similar and do exhibit dips consistent with the multipath pattern in Figure 10, as per the formulations in (4) and (9). The nulls are likely filled because of the noise floor and the presence of RF interference (RFI). The latter apparently was rather ubiquitous with the site being near an active airfield and could have imparted slight kinks over the sphere response curves even away from the nulls.

Before applying Formulas (3)–(9) to determine the ground reflection coefficient $\left|\Gamma_{g}\right|$ (the principal parameter governing the multipath pattern), we need to refine the measurement for the antenna height $h_{A}$ that also enters the equations. While we were able to measure the height of the antennas on the truck above the road surface as 3.25 m, this is not the same as the height above the target plane used in the model, cf. Figure 9. For one thing, the road was somewhat raised over the surrounding fields, but this was not reflected in the available digital elevation models (the step in the Google Earth elevation data that we checked was 1 m). Formulas like (10) and (11) indicate that the relative accuracy of the result would be a combination (in the root mean square sense) or the relative accuracies of the individual factors. With the antenna elevation being of the order 3–4 m, even a half-meter error in this parameter introduces a 12% error in the null location predictions. In comparison, the target heights, though smaller, are measured with at least 1 cm accuracy, which, in relative terms, would be 2.5% for the lowest target.

**Figure 12:**
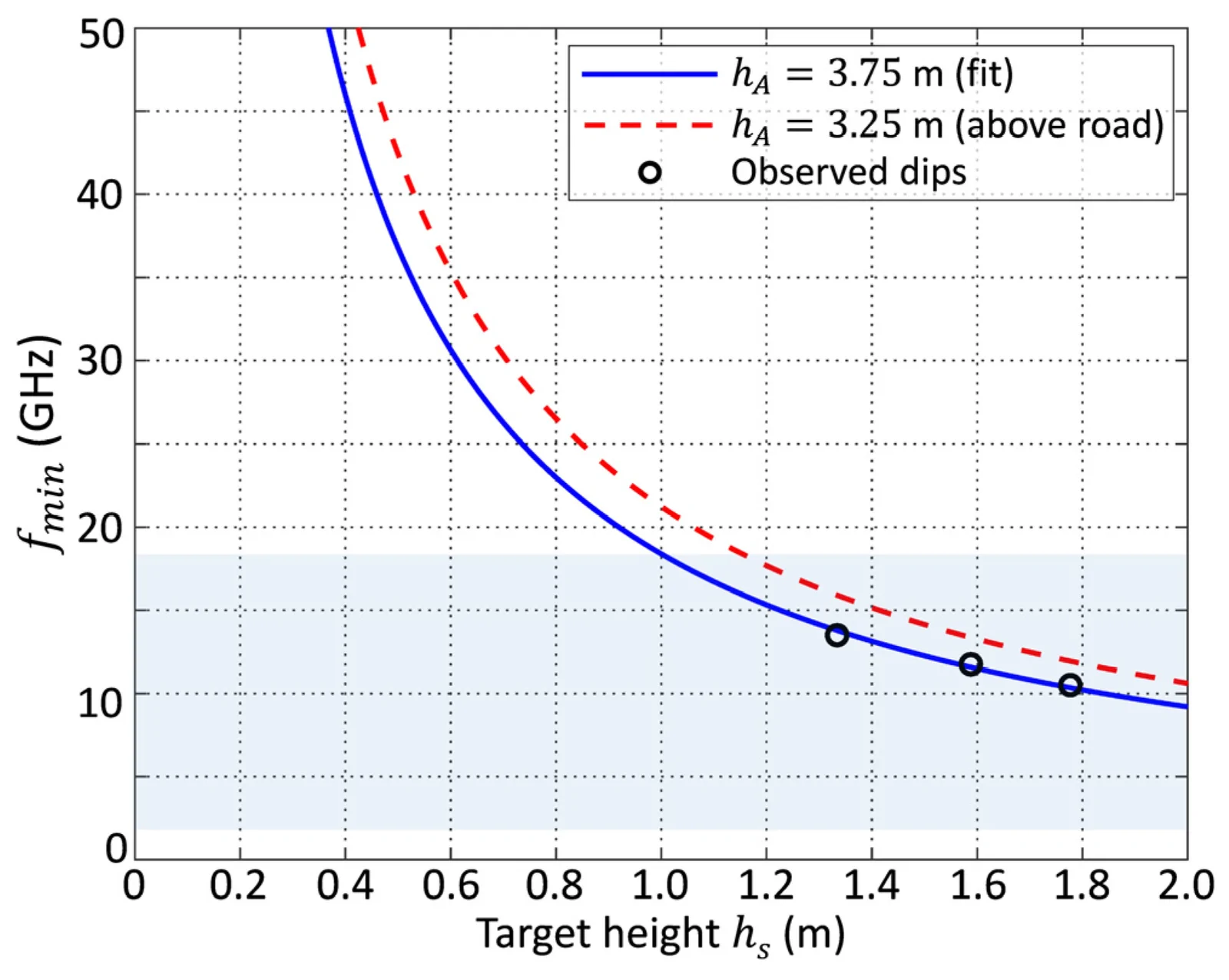
Behavior of the first non-trivial null of the multipath pattern vs. target elevation and determination of actual antenna height above the common plane by best theoretical fit. The shaded area indicates the frequency range of the system.

We proceeded with estimating the locations of the nulls in Figure 11 and finding the value of $h_{A}$ that gave the best match with the predictions from Equation (11) (with n=1). Specifically, we visually determined the null locations to be 13.5 GHz, 11.75 GHz, and 10.5 GHz for the 1.8 m, 1.6 m and 1.3 m curves, respectively (we tried to make the best judgment based on both VV and HH curves, assuming that at low grazing, the two polarizations should have been affected by the multipath pattern in the same way). As illustrated in Figure 12, the best fit is achieved with $h_{A}=$ 3.75 m, while the curve corresponding to “above-the-road” antenna height measurement of 3.25 m is considerably off. Determining the location of broad filled nulls in the presence of kinks is admittedly challenging. Yet the outcome for $h_{A}$ is rather stable. For example, if the dip location for the 1.3 m sphere (the most difficult case) was to be taken as 14 GHz (shifting its marker in Figure 12), the resulting $h_{A}$ would change to 3.69 m. It is $h_{A}$ rather than individual null locations that contributes to the subsequent analysis.

Finally, to estimate $\left|\Gamma_{g}\right|$ from the measured data $S_{meas}^{sphere}$, one needs to normalize out the system part of the response, $T_{system}\left(f\right)$, cf. (3), (5). This can be done by dividing the elevated target responses by that of the target in the lowest position ($h_{s0}=$ 0.4 m) that conveniently does not feature any nulls:

(12) $$Q\left(f,h_{s},h_{s0},\left|\Gamma_{g}\right|\right)=\frac{{\left|T_{mp}\left(f,h_{s},\left|\Gamma_{g}\right|\right)\right|}^{2}}{{\left|T_{mp}\left(f,h_{s0},\left|\Gamma_{g}\right|\right)\right|}^{2}}=\frac{{\left|S_{meas}^{spher}\left(f,h_{s}\right)\right|}^{2}}{{\left|S_{meas}^{spher}\left(f,h_{s0}\right)\right|}^{2}}.$$

The right-hand side is formed from actual measurements (shown in Figure 11) while the ratio on the left comes from (9) using the proper values of the target height. Ideally, the two are expected to be equal, but in practice, the measured data are affected by noise, RFI and other uncontrollable factors. To reflect that, we will label the ratio on the right as $Q_{meas}$. We estimate the reflection coefficient by finding the best fit of the theoretical $Q\left(f,h_{s},h_{s0},\left|\Gamma_{g}\right|\right)$ to the measured quantity over the available frequency points:

(13) $$\hat{\left|\Gamma_{g}\right|}=\underset{\left|\Gamma_{g}\right|}{\mathrm{argmin}}\sum_{i}{\left|Q\left(f_{i}\right)-Q_{meas}\left(f_{i}\right)\right|}^{2}.$$

**Figure 13:**
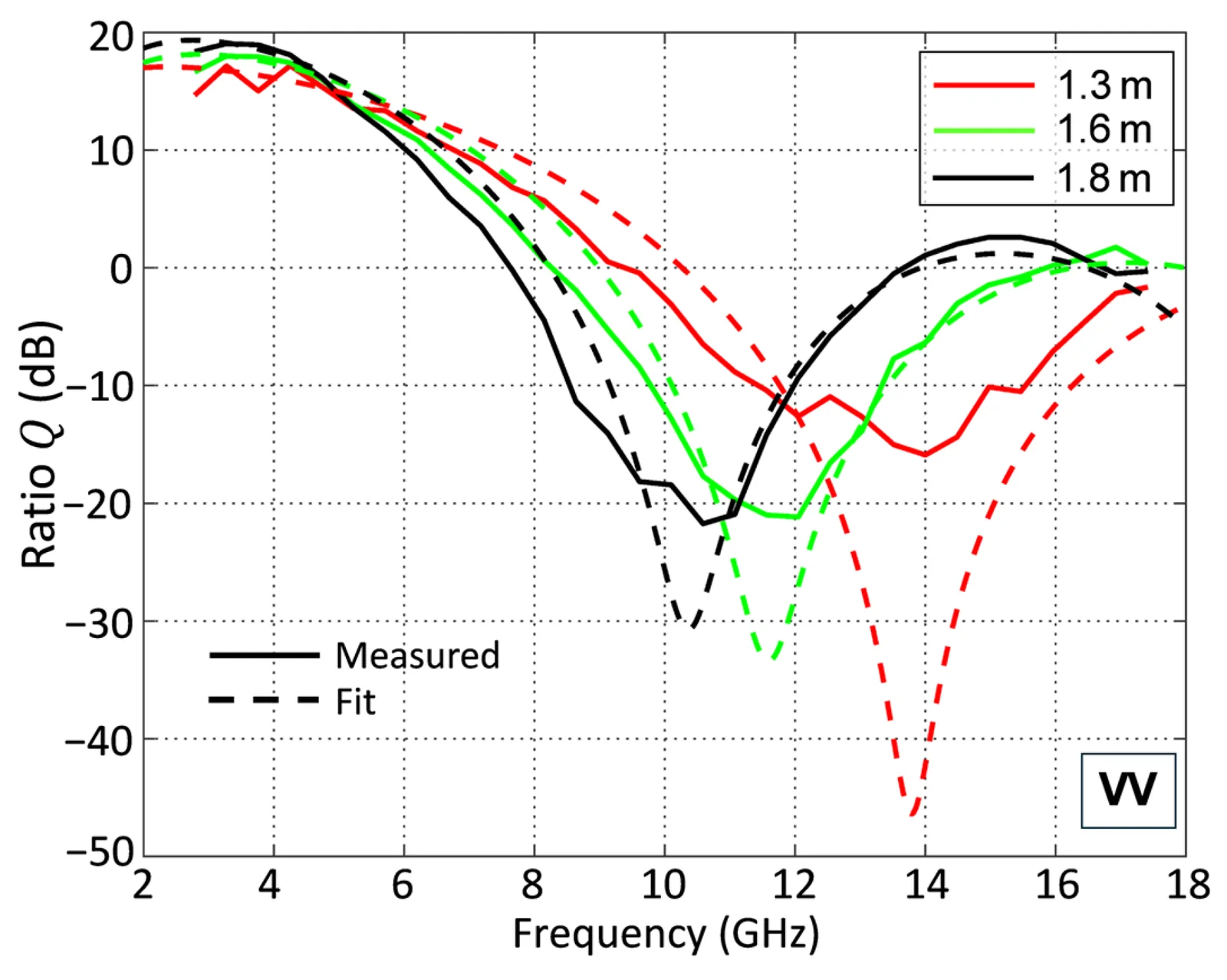
An example of the measured ratios Q in Equation (12) (solid lines) and the best fits (dashed lines) for the three higher target positions. VV polarization case.

**Figure 14:**
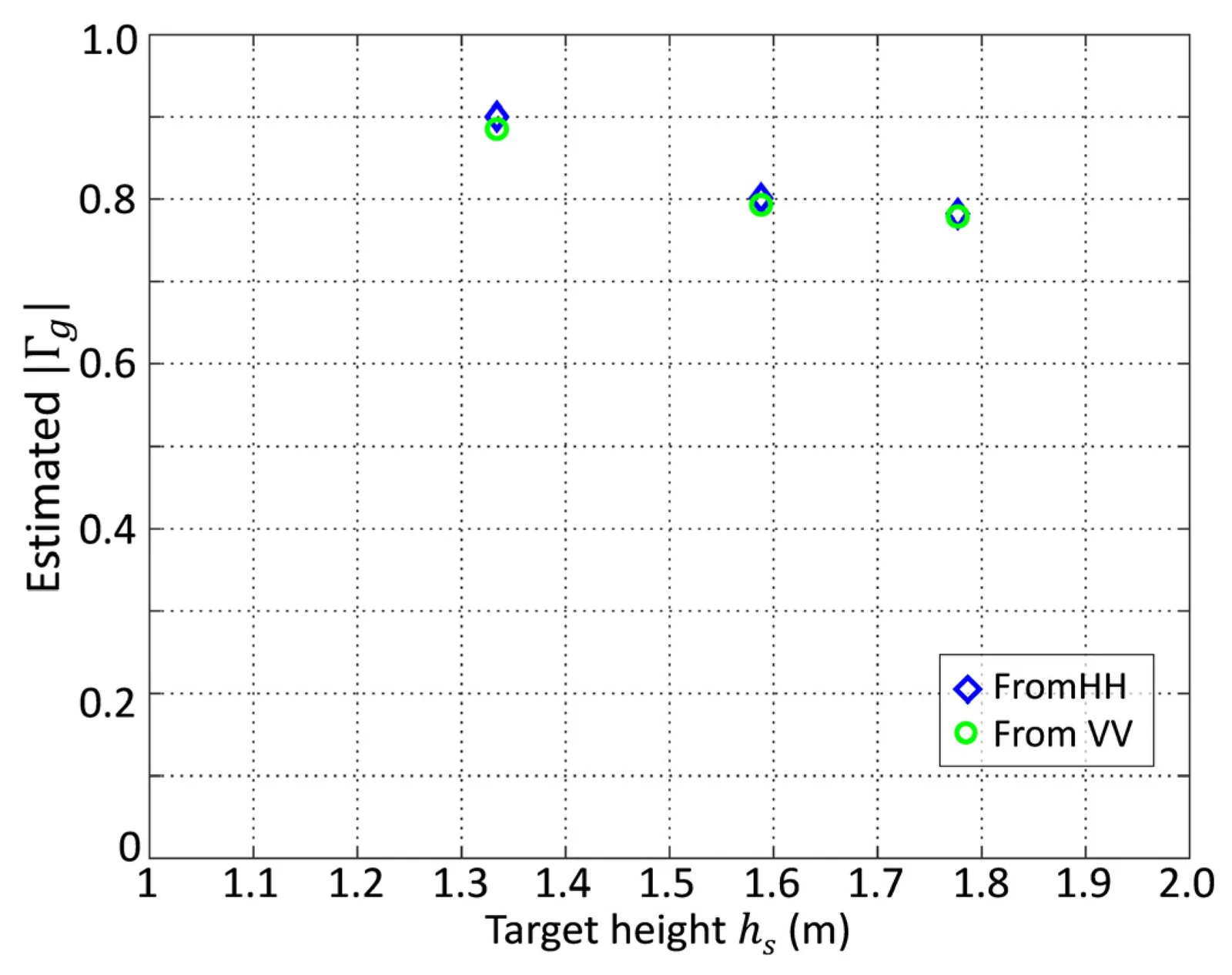
Estimated absolute values of the reflection coefficient using Equations (12) and (13).

As (13) shows, we use the quantities in the natural units rather than in dB (the latter would accentuate the nulls where the accuracy is in fact low). The edge data points were excluded because of the low values of the numerator and concerns over RFI contamination. An example of such a fit is given in Figure 13 for the VV polarization; the HH plots are very similar. The estimated values of the reflection coefficient are shown in Figure 14.

It is observed that the values retrieved from the VV and HH backscatter are rather close. There is, however, a noticeable difference between the values for the highest two sphere positions and those for the 1.3 m position—0.8 vs 0.9, respectively. While we do not have a definitive explanation for this behavior, we observe that the multipath reflection points move farther away from the target location as the latter height increases, cf. Figure 9. The reflections for the lowest two heights occur about 50 and 120 m away, staying within the field in the bottom (southern) part of Figure 5. For the two highest positions, the estimated specular-point locations are 137 and 148 m, putting them into a different land parcel that is apparently managed in a separate way and has a different texture in satellite images. This could be a possible reason for the differences in $\left|\Gamma_{g}\right|$ observed in Figure 14. If one follows this hypothesis, there could be another iteration in estimating the reflectivity for the two highest target elevations. The $T_{mp}$ in the denominator in Equation (12) (multipath pattern for the lowest position) should be supplied with the value of $\left|\Gamma_{g}\right|$ determined for the 1.3 m height, as the reflections for the two occur on the same surface type. This, however, leads to a less than 2% change to the initial estimates shown in Figure 14.

For a completely smooth surface (taking the soil dielectric permittivity to be, say, 3) and near-grazing angles (0.6−0.7°), theoretical values for $\Gamma_{g}$ would be −0.95 or −0.98, depending on polarization, see [26]. The results in Figure 14 indicate smaller absolute values, especially for the two higher targets. Possible reasons could be surface roughness and the presence of vegetation causing the scattering and attenuation of the multipath contribution. The reduction of $\left|\Gamma_{g}\right|$ due to rough surface scattering can be modeled by multiplying the ideal, smooth-surface reflection coefficient by the “Ament factor”,

(14) $$F_{A}=exp\left\{-2{\left(\frac{2\pi fH_{rms}}{c}\sin\psi_{gr}\right)}^{2}\right\},$$

where *H_rms_* is the root mean square of the surface roughness, and $\psi_{gr}$ is the grazing angle [27]. For our experimental configuration, we take $\psi_{gr}≅0.7{}^{\circ}$ and estimate that *H_rms_* < 1 cm. This results in $F_{A}=0.99$ in the most challenging case of the highest frequency (largest k in the formula in Equation (14)). Thus, $F_{A}$ is very close to 1 across the entire SKuSAR band, indicating that the determined relatively low value of the apparent reflection coefficient, $\left|\Gamma_{g}\right|=0.8$, cannot be attributed to surface roughness alone. Attenuation and scattering due to grassy vegetation are likely to be the primary reasons for the reduction in the value of $\left|\Gamma_{g}\right|$.

**Figure 15:**
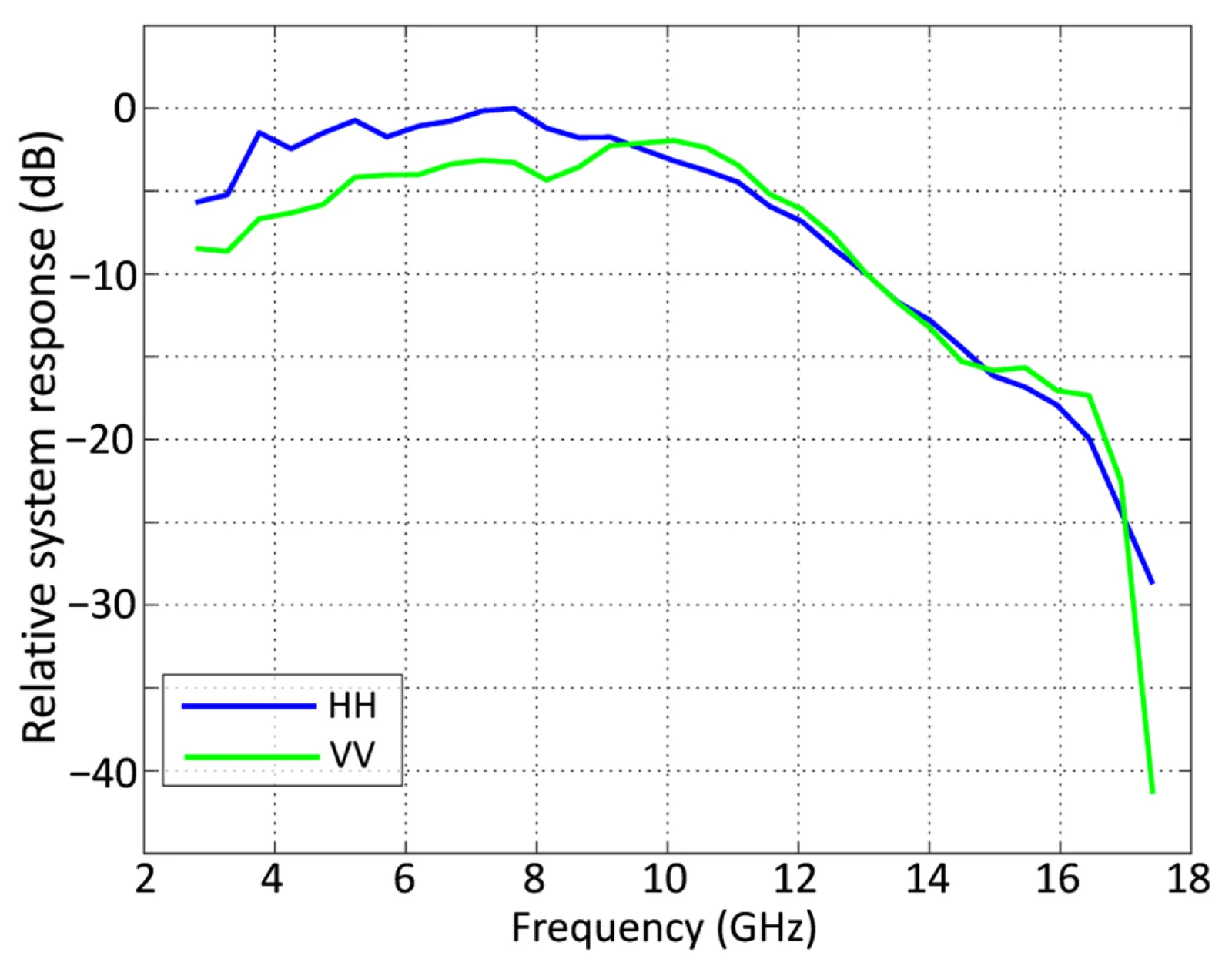
Estimated SKuSAR system response ${\left|T_{system}\right|}^{2}$ for HH and VV polarizations (normalized to the highest common value).

With the multipath characteristics determined, we can attempt to estimate the system response, as defined in (5). The absolute values are shown in Figure 15 for both VV and HH polarizations, with the plots normalized to a common maximum. There are some noticeable differences of a few dB between the two for frequencies below 9 GHz. This is something that is also manifested in the VV and HH corner reflector signatures in Figure 7a. The response curves then coalesce and drop as the frequency grows. This reduction is due to the decrease in the AWG output power with frequency, particularly above 14 GHz, and frequency-sensitive loss in the antenna cables (combined length of 15 m), which increases from 0.2 dB/m at 2 GHz to 0.68 dB/m at 18 GHz. In principle, such joint system response curves can form the basis for polarimetric calibration across the frequency domain. However, the multipath reflections introduce obvious uncertainties and complications. It has therefore been deemed prudent to defer the full-scale calibration efforts to airborne deployment when multipath effects, if any, would be range-resolved.

## 4. System Resolution and Point Target Data

As stated in the Introduction, our focus in pursuing the development of the system is to exploit target and scene variations that may occur across SKuSAR’s multiple octaves of bandwidth (getting the “spectral signatures”). Still, the ability to produce potentially ultra-sharp (at least in range) images using the entire available band is intriguing and expedient. A thorough analysis of the range–azimuth impulse response function of a SAR system is given, for example, in [28]. Here, we discuss the full-band (as well as sub-band) target image dimensions seen with our instrument and highlight some issues that could affect image fidelity for UWB radars. For the resolution assessments, the images were processed with 1 cm sampling in azimuth and 2 mm in ground range. When finding widths, range and azimuth cuts were interpolated to a 0.5 mm grid.

The well-known rule of thumb for determining the spatial resolution of a radar in range $\delta_{r}$ is

(15) $$\delta_{r}=\frac{c}{2B}$$

(cf. also Equation (A8)). Usually, this is expected to be the width of the response from a “point target”. Notably, while none of our calibration targets qualify as “point” one based on the physical size for the full-band 1 cm resolution predicted by (15), both a trihedral and a sphere should behave as virtual point scatterers positioned at their apex and geometrical center, respectively. In the raw imagery (before speckle-reduction smoothing), we observed the range response for the trihedral in the center of Figure 4a to be about 3.1 cm (measured at half-amplitude or a −6 dB power level). This is more than the expected ~1.8 cm value that accounts for applying the Hamming window. Also, despite the window, the nearby sidelobes considerably exceed the window-dictated -43 dB level. In particular, the first sidelobe on the “far range” side is about −20 dB high. We believe this to be mainly due to the multipath effect: the one-way range associated with the single-bounce path depicted in Figure 9 is about 1.5 cm longer than that for the direct path. The weaker two-bounce path (last term in Equation (6)) with its 2.9 cm excess range could acquire increased prominence at higher frequencies as the bistatic reflection pattern of the trihedral sharpens. Variations in the system response could also be causing some broadening.

Even for a well-calibrated UWB system and absent multipath effects (specific to our near-grazing observation geometry), other fundamental factors can be at play. Across the available spectrum, a target assumes a vastly different electromagnetic size (physical dimension normalized by wavelength), which affects its scattering properties and makes the backscattering amplitude frequency-dependent. For example, at 2 GHz, the 20-inch sphere is still in the Mie scattering regime, moving into the optical region as the frequency increases [20] (p. 34) and [29]. If we consider a true “point” physical target (e.g., a sphere with dimensions much smaller than the 1 cm resolution), it will be of a sub-wavelength size for the entire frequency band. Consequently, it will behave as a Rayleigh scatterer with the cross section proportional to $f^{4}$ and the scattering amplitude, respectively, behaving as $\sim f^{2}$ [29]. Such a quadratic frequency ramp across the received signal spectrum (adding the second derivative of the envelope in the time/range domain) can result in a main lobe distortion and increased sidelobes. In this example, the phase of the scattering amplitude does not change, but such variations could also be an aggravating factor for signal compression performance. For a trihedral (operating in the geometrical optics mode), the cross section grows as $f^{2}$, while the scattering amplitude is proportional to f [30].

The notion of resolution still remains an intrinsic property of the UWB system (e.g., Appendix A shows that the range resolution of the FMCW radar is conceptually tied to the corresponding characteristic of the Fourier spectrometer). In particular, this parameter will still describe the correlation distance of intensity fluctuations when imaging distributed scenes—the knowledge being essential for devising and assessing speckle-reduction filters. On the other hand, such a system, even when perfectly calibrated, is not guaranteed to produce a clean undistorted target image exactly consistent with the stated resolution. The reason lies with the frequency-dependent scattering properties of physical targets that are beyond control of the system and its operators. Incidentally, it is such variations that the sub-band approach aims to use for target and scene characterization.

In discussing azimuth resolution, [28] considers a fixed “integration angle”, which would be consistent with beam-scanning, spotlight-mode image formation. For the broadside-looking SAR, this angle is defined by the antenna pattern that can vary from tens of degrees at the lower frequency end to just a few degrees on the Ku side of the spectrum. A windowing function often applied during processing could have different “beam-limiting” effects at lower and higher frequencies. The resolution formula for a narrowband side-looking SAR operating at central frequency f (with the wavelength λ=c/f) is

(16) $$\delta_{a}=\frac{\lambda R}{2L}=\frac{c}{4f\tan(\Delta \theta /2)},$$

where R is the slant range to the target, and L and Δθ are, correspondingly, the effective synthesized aperture width and the effective azimuth beamwidth. The latter two can be entirely defined by the antenna pattern or result from weighting during processing. The antenna pattern width, in turn, can be related to the azimuth aperture extent $D_{a}$ as (e.g., [31])

(17) $$\sin(\Delta \theta /2)≅c/\left(2fD_{a}\right).$$

Combining the two equations results in

(18) $$\delta_{a}=\frac{D_{a}}{2}\sqrt{1-{\left(\frac{c}{2fD_{a}}\right)}^{2}}=\frac{D_{a}}{2}\sqrt{1-{\left(\frac{\lambda }{2D_{a}}\right)}^{2}}$$

When $\lambda \ll D_{a}$ (this condition perhaps could be relaxed to $\lambda <D_{a}$), Equation (18) leads to

(19) $$\delta_{a}≅D_{a}/2.$$

**Figure 16:**
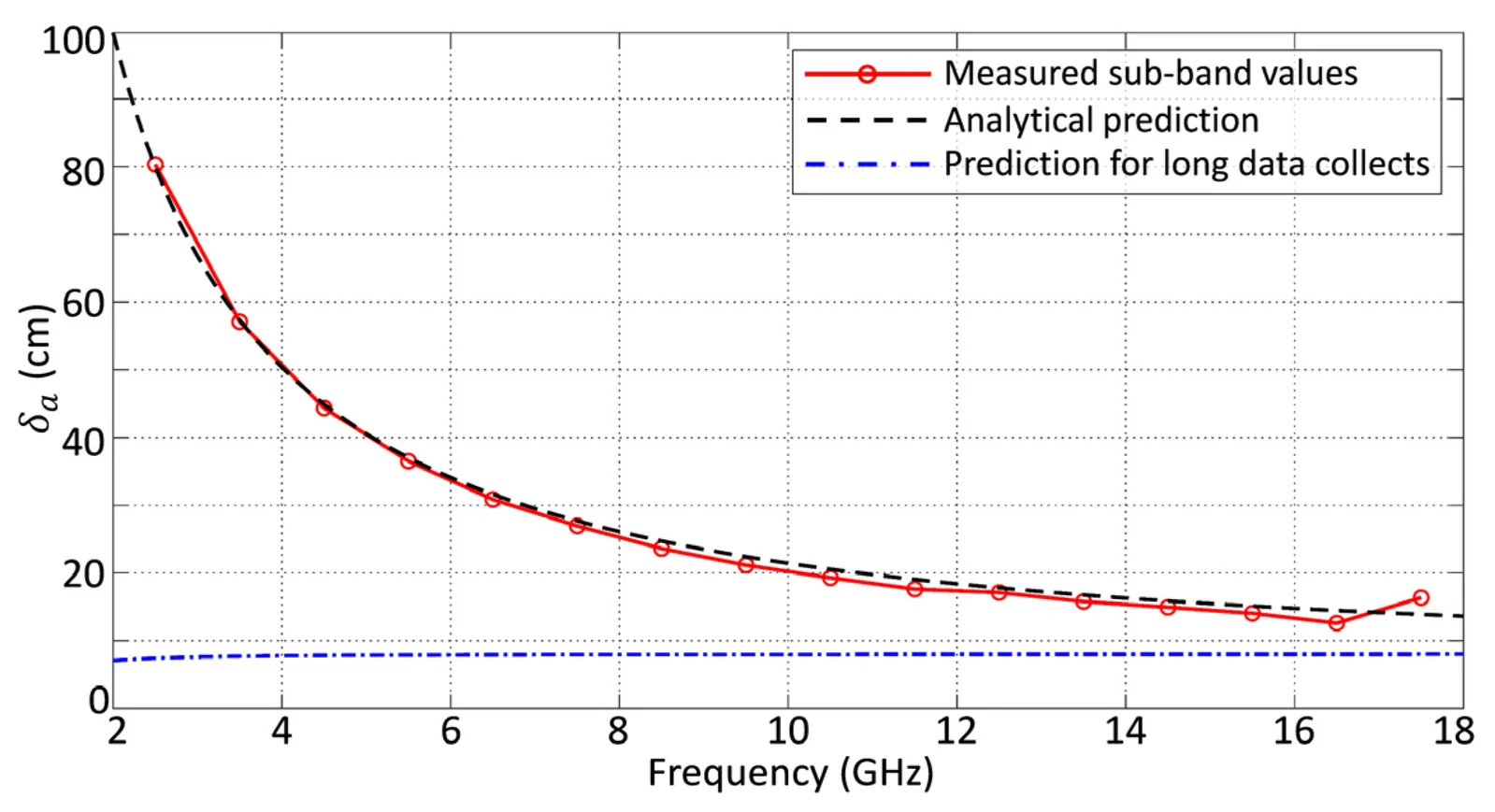
Narrowband azimuth resolution vs. frequency as given by Equation (20) (dashed line) and the values measured for sub-band images (solid line with markers). The azimuth resolution predicted by Equation (18) (i.e., no limits on the data segment duration) is shown for reference with a dash-dotted line.

The latter is a well-known formula in the SAR theory, usually derived under the narrow-beam assumption, e.g., [14] (strictly speaking, to employ Equations (16) and (18) with large aperture angles, one would need to consider tapering effects of range loss as well). Based on purely geometrical sizes, the equivalent transmit–receive aperture in our case would be about 26 cm, which should be applicable to the HH-polarized signals. For the VV polarization example that we are considering, the effective aperture width further reduces to 16 cm due to the H-plane field tapering in the horns. In both cases, Equation (19) generally holds across the system band. The largest relative error occurs at 2 GHz and is less than 5% for the 26 cm aperture, rising to 13% for its 16 cm counterpart (for the latter case, the expected behavior of $\delta_{a}$ in Equation (18) is shown in Figure 16).

If we view the response of the UWB SAR as a superposition of the narrowband constituents, the frequency-independent quantity in Equation (19) should largely apply to the resulting system response as well (possible phase variations across the azimuth resolution cell, although not expected in this case, could have a modifying effect.) That is, we regard the quantity in (19) as the target figure for the azimuth resolution for our UWB broadside-looking SAR, and for the VV polarization $\delta_{a}$ thus could be as fine as 8 cm. However, our truck-based collections had limited spans of about 67 m (with the trihedral being at the center of the synthesized aperture), and during the processing the entire data segment was weighted by the Hamming window. With this additional tapering, the resulting narrowband azimuth resolution could be approximated as

(20) $$\delta_{aw}≅\sqrt{\delta_{a}^{2}+\delta_{w}^{2}},$$

with $\delta_{a}$ given by (19), and $\delta_{w}$ being the resolution in (16) due only to the Hamming taper. In the latter case, the effective width would be 0.54 × 67 m = 36.2 m. The formula is obtained by approximating the weighting functions by Gaussians retaining the same effective widths, e.g., [32,33] and evaluating the width of their product. The quantity $\delta_{w}$ would be frequency-dependent—as well as the resulting $\delta_{aw}$. For the trihedral distance of 480 m, the formula in (20), together with (16) and (19), yields $\delta_{aw}=$ 13.6 cm at 18 GHz and 100 cm at 2 GHz. The azimuth resolutions measured for our 1 GHz sub-band responses show rather good agreement with the analytical prediction, cf. Figure 16. The corresponding range resolutions for the sub-bands are around 27 cm, consistent with the bandwidth and the applied Hamming weighting. The full-band azimuth resolution for our UWB SAR is measured to be 23.6 cm. Once again, this number is affected by the finite length (duration) of the data segment inherent to our truck-based setting. For the airborne case with no such limitations, the full-band azimuth resolution is expected to be closer to what is predicted by (19).

## 5. Discussion and Conclusions

In this paper, we describe the SKuSAR system, a 2–18 GHz FMCW SAR designed as a test bed for the development of new remote sensing algorithms that exploit variations in the scene response across polarization and the system’s ultra-wide bandwidth. The system is composed of commercial, off-the-shelf components, and features a 2–18 GHz arbitrary waveform generator. The truck-based deployment early into the program allowed the testing and perfection of various aspects of the system hardware and its performance. It provided much-needed real data for adapting and fine-tuning the existing SAR processing code to the FMCW operation. The observed resolutions were consistent with the expectations. Finally, the field work allowed the demonstration of various concepts such as sub-band processing and system response calibration.

We were also successful in adapting a standard back-projection-based SAR processor (previously used with our other pulsed systems) for the UWB FMCW data. We were encouraged that the possibility had been discussed in the literature, e.g., [34]. It was found, however, that the qualitative and intuitive description of the FMCW-based ranging outlined in Section 2.2 (and going back to Skolnik’s book [20]) was not sufficient and there were other additional operations to be performed. The consistent mathematical treatment of the FMCW range compression based on the correlation principle is given in Appendix A. The resulting expressions encompass the procedural steps described in Section 2.2 and reveal the phase factors to be applied. The latter has profound impacts on the success of SAR azimuth synthesis. The expressions can be generalized to describe a sub-band FMCW processing with arbitrary bandwidth, thus giving a mathematical foundation to the multispectral processing.

Splitting the total bandwidth into sub-bands is an interesting and consequential problem that generally needs to weigh the benefits of achieving finer spectral resolution against suffering reduced signal-to-noise ratios. In this study, we concentrated on the response of bright calibration targets and found empirically that working with 1 GHz sub-bands was acceptable on both accounts. An in-depth analysis of the issue with a focus on ocean surface backscatter is given in [9]. For a set sub-band width, one can also consider a smaller stride to get smoother spectral signatures—although at the expense of higher processing loads.

Despite offering an economical testing solution, the ground-based setting had some complicating peculiarities. One was the presence of a strong multipath component in the calibration target signatures. While often a common occurrence in ground-based radar systems, e.g., [35], this phenomenon was not a factor in our past airborne campaigns and took some time to properly identify and analyze. In the process, it was shown that the multispectral approach could be instrumental in determining the electromagnetic properties of the reflecting ground. For the main task of calibrating the system’s spectral response, the multipath mitigation strategy (minimizing the target’s height above the ground) was adopted. Another distinctive attribute of the truck-based SAR was the rather limited duration of the data segment, which affected the extent of the synthesized aperture. The reasons included both the feasibility of the traffic pattern and local line-of-sight obstructions. This finite data extent had larger impacts at lower frequencies where the antenna patterns became increasingly broad. The corresponding variations in the narrowband SAR azimuth resolution were examined with the sub-band approach and showed good agreement with the theoretical predictions. While these short data span-related effects prevented the system from achieving the expected full-band azimuth resolution stated in (19), the agreement with the theory is very encouraging. As for range resolutions, both the sub-band and the full-band values were close to expectations, although near-grazing multipath did exert some impact.

We emphasize that these complicating factors, while having required close attention here, are not expected to be present or significant in the airborne data once the radar flies on the Navy UV-18 (Twin Otter) aircraft. For example, the single-bounce multipath delay for a tripod-mounted calibration target is expected to be about 90 cm—large enough for the echo to appear in a different range resolution cell even with the sub-band processing. The magnitudes of the single- and double-bounce signals will be further reduced due to lower values of Fresnel reflection coefficients at intermediate incidence angles [26]. An aircraft could follow a straight track for a sufficiently long distance to make the encountered limited data extent effects a non-issue. It should be noted that the radar in the ground-based experiment largely operated with the settings planned for the airborne campaign. The pulse repetition interval of 248 μs yields the per-channel PRF of 2.0 kHz (accounting for alternating V and H transmissions) and is more than enough to accommodate the expected Doppler bandwidth of ~713 Hz during flights. The latter number was obtained using an aircraft speed of 57 m/s (110 knots) and the antenna half-pattern width in Equation (18). The range gate extent is “hard-wired” through the IF filter and would remain between approximately 440 m and 580 m, as described in Section 2.1. The range loss therefore will be the same as in the truck experiment. With the envisaged flight altitude of 229 m (750 ft), this would translate into the span of incidence angles from 59° to 67° and the 157 m wide ground swath (not all of it may be illuminated by the narrowing antenna pattern at the upper end of the frequency band). At these angles, the extended scenes like fields or water bodies should be much brighter (compared to what is seen in Figure 4), as the normalized radar cross section for rough surfaces increases rapidly away from near-grazing incidence [36]. On the other hand, for an airborne system the calibration (as well as data interpretation in general) may be challenging if significant roll or other attitude changes occur, as a swinging antenna pattern—that is rather narrow at higher frequencies, being ~4–6° at 18 GHz—would need to be tracked and accounted for (stabilized antenna positioners should solve such a problem). As for the SAR image formation itself, the back-projection processor with its intrinsic motion compensation capabilities should work just as well, with the positions of the transmit/receive phase centers accurately tracked by the INS.

There are more multispectral descriptors, as well as calibration-related tasks, that have not been considered or attempted during this demonstration system deployment. Besides the magnitude, the phase of the across-the-band frequency responses (such as in Figure 7, Figure 11 and Figure 15) is also of interest. To effectively utilize polarization capabilities of the system, full polarimetric calibration (that also involves phase) is required. The procedure is expected to be carried out on a sub-band basis—cognizant of the phase issues that can arise over multi-octave bandwidth [37]. Spectral signatures of non-canonical objects and extended scenes will be of interest—to demonstrate, explore and advance the multispectral sensing premise for this UWB SAR sensor. Future airborne deployments should provide suitable high-quality data to proceed with these studies.

## Data Availability

The raw full-resolution data are restricted because of organizational and government regulations. Requests should be directed to the Commanding Officer, Naval Research Laboratory, Washington, DC 20375, USA. The reduced-resolution imagery used in this article may be available upon request by contacting the corresponding author.

## Abbreviations

The following abbreviations are used in this manuscript:

AWG	Arbitrary waveform generator
BP	Back projection
DAS	Data acquisition system
FMCW	Frequency-modulated continuous wave
IF	Intermediate frequency
IMU	Inertial measurement unit
INS	Inertial navigation system
LFM	Linear frequency modulation
LO	Local oscillator
PRF	Pulse repetition frequency
PRI	Pulse repetition interval
RCS	Radar cross section
RF	Radio frequency
RFI	Radio frequency interference
SAR	Synthetic aperture radar
SNR	Signal-to-noise ratio
UWB	Ultra-wideband

## Symbols

The following symbols are used in this manuscript:

$f_{L}$$,\;f_{U}$	Start and end frequency of the FMCW waveform	Figure 3, (A1), (A5)
Δf	Propagation incurred frequency shift between the transmitted and received signals	Figure 3, (1)
$T_{chirp}$	Sweep time of the FMCW waveform (PRI)	(2)
$B_{tot}$	Total system bandwidth	(2), (A5)
α	LFM chirp rate	(2)
f	Electromagnetic frequency	
$S_{meas}$	Target response	(3)
$T_{system}$	System transfer function	(3)
$T_{mp}$	Multipath factor	(4)
$h_{s}$	Height of targets (sphere) above ground	Figure 9, (4)
$h_{s0}$	The lowest above-ground height of the sphere	(12)
$h_{A}$	Height of the radar antenna above ground	Figure 9, (8)
$R_{0},\;R$	Target distance to radar along the slant range	Figure 9, (1), (6), (A2)
$R_{g}$	Target distance to radar on the ground plane	Figure 9, (7)
$R_{1}$	Range distance of the ground-bounced path	Figure 9, (8)
$\Gamma_{g}$	Forward reflection coefficient of the ground	(6)
c	Speed of light in air	(6)
$f_{min}$$,\;f_{max}$	Frequencies corresponding to null response and peak response	(10), (11)
Q	Ratio of the scattered powers from targets (sphere) at two different heights	(12)
$H_{rms}$	Root mean square of the ground surface height variation	(14)
$\psi_{gr}$	Grazing angle	(14)
$F_{A}$	Ament factor: scaling on the forward reflection ratio of the ground due to surface roughness	(14)
$\delta_{r}$	Slant range resolution	(15), (A8)
$\delta_{a}$	Azimuth resolution	(16)
L	Synthetic aperture length	(16)
Δθ	Beamwidth of azimuth beam integration	(16)
$D_{a}$	Physical aperture size of the antenna in azimuth	(17)
λ	Wavelength corresponding to electromagnetic frequency f	(16)
$s_{tx}$	Transmitted signal	(A1)
$w_{T}$	Rectangular window of width T	(A1)
$s_{rx}$	Received signal	(A2)
a	Amplitude of the received signal	(A2)
p	Correlation of the received signal with its expected copy	(A3)
$s_{ref}$	Reference signal	(A3)
r	Range parameter in the reference signal	(A3)
P	Complex envelope of the correlation p	(A4)
$f_{c}$	Center (carrier) frequency	(A5)
Δr	Slant range bin size	(A7)
$\Delta f_{FFT}$	FFT frequency step	(A7)
T	Interval over which Fourier transform is taken	(A7)
$r_{max}$	Maximum unaliased range	Appendix A
$\Delta t_{s}$	Sampling interval of the IF signal	Appendix A
$P_{sub}$	Complex envelope of the correlation for sub-band processing	(A9)
$w_{T_{sub}}$	$\mathrm{Rectangular}\;\mathrm{window}\;\mathrm{of}\;\mathrm{width}\;T_{sub}$	(A9)
$f_{csub}$	Center frequency for a sub-band	(A9), (A10)
$f_{l}$$,\;f_{u}$	Lower and upper frequencies for a sub-band	(A10)
$T_{sub}$	Chirp duration corresponding to a sub-band	(A11)
$B_{sub}$	Sub-band bandwidth	(A11)
$t_{csub}$	Temporal center of a sub-band chirp	(A11)
$\delta_{r\;sub}$	Slant range resolution corresponding to a sub-band	(A12)

## Appendix A

Here, we provide a mathematical treatment of the FMCW range, considering it as a correlation processing problem. The transmitted FMCW signal within one period starting at t=0 and ending at $t=T_{chirp}$ can be expressed as

(A1) $$s_{tx}\left(t\right)=w_{T}\left(t-T_{chirp}/2\right)\exp\left(j\pi \alpha t^{2}+j2\pi f_{L}t\right).$$

Above, $w_{T}(t)$ is a rectangular window of the extent $T_{chirp}$ centered around 0 (hence the shift in the argument), while $f_{L}$ and α are, respectively, the lower end of the entire frequency band and the chirp rate, as introduced in Section 2.2. In the truly continuous-signal system, the pulse repetition interval should be the same as $T_{chirp}$, but it is not required and is not relevant. The received signal from a point target at range R would be

(A2) $$s_{rx}\left(t,R\right)=as_{tx}\left(t-2R/c\right).$$

The formula in (A2) ignores any frequency dependence of the target scattering amplitude a. As a general principle, one should evaluate correlations of the received signal with its “reference copies” that presumably emanate from all possible ranges r, with the maximum indicating the actual target position:

(A3) $$p\left(r,R\right)=\int_{-\infty }^{\infty }s_{rx}\left(t,R\right)s_{ref}^{*}\left(t,r\right)dt.$$

The function $s_{ref}$ in (A3) is given by (A2) with the argument replacement R→r and the amplitude a set to 1. The correlation output p still contains the “carrier frequency” component (oscillating in variable r—the second argument R now serves as a parameter labeling the location of the actual target). One takes its complex envelope

(A4) $$P\left(r,R\right)=p\left(r,R\right)e^{-j2\pi f_{c}(2r/c)}$$

as this is what conventional pulse compression techniques normally yield. The “carrier” or center frequency $f_{c}$ above is defined as

(A5) $$f_{c}=\frac{f_{L}+f_{U}}{2}=f_{L}+\alpha \frac{T_{chirp}}{2}\equiv f_{L}+\frac{B_{tot}}{2},$$

with $f_{U}$ being the upper end of the system frequency band and $B_{tot}$ being the total bandwidth. Upon substituting the explicit expression for the reference signal into (A3) and noting that range-related delays should be much smaller than the FMCW chirp duration, we can write the resulting complex envelope as

(A6) $$P\left(r,R\right)=\exp\left\{-j\pi \alpha T_{chirp}\frac{2r}{c}\right\}\exp\left\{-j\pi \alpha {\left(\frac{2r}{c}\right)}^{2}\right\}\;\times \int_{-\infty }^{\infty }\left[s_{rx}\left(t,R\right)w_{T}\left(t-T_{chirp}/2\right)\exp\left(-j\pi \alpha t^{2}-j2\pi f_{L}t\right)\right]\exp\left\{j2\pi \alpha \frac{2r}{c}t\right\}dt.$$

The expression in the square brackets under the integral corresponds to the mixing operation with the transmitted (or LO) signal, cf. Equation (A1), as described in Section 2.2. The product then undergoes Fourier transform, with the combination α2r/c playing the role of frequency, cf. (1). For discretized signals, (A6) can be evaluated by FFT over some interval $T\geq T_{chirp}$ (i.e., with possible zero-padding), with the resulting range bin size related to the corresponding FFT frequency step $\Delta f_{FFT}=1/T$ as

(A7) $$\Delta r=\frac{c}{2\alpha }\Delta f_{FFT}=\frac{c}{2\alpha T}.$$

The *resolution* in frequency, and hence in range, following the transform is dictated by $T_{chirp}$:

(A8) $$\delta_{r}=\frac{c}{2\alpha }\delta_{f}=\frac{c}{2\alpha T_{chirp}}=\frac{c}{2B_{tot}},$$

cf. Equations (A5) and (2). Other useful parameters can be determined similarly by invoking familiar concepts from time–frequency analysis. For example, the “Nyquist range” for unaliased measurements would be related to the sampling interval of the mixed IF signal as $r_{max}=c/\left(4\alpha \Delta t_{s}\right)$.

Note that the pulse compression operation in (A6) involves multiplications by complex exponentials appearing in front of the integral. These factors, especially the first one, are essential for the subsequent SAR azimuth compression to succeed. As the latter operation in BP requires the “central frequency” parameter, the value of $f_{c}$ in (A5) is supplied.

One can demonstrate in a similar fashion that the compression procedure for a sub-band signal with the LFM ramp stretching from $f_{l}$ to $f_{u}$ can be formulated as

(A9) $$P_{sub}\left(r,R\right)=\exp\left\{-j\frac{4\pi }{c}(f_{csub}-f_{L})r\right\}\exp\left\{-j\pi \alpha {\left(\frac{2r}{c}\right)}^{2}\right\}\;\times \int_{-\infty }^{\infty }w_{T_{sub}}\left(t-t_{csub}\right)\left[s_{rx}\left(t,R\right)\exp\left(-j\pi \alpha t^{2}-j2\pi f_{L}t\right)\right]\exp\left\{j2\pi \alpha \frac{2r}{c}t\right\}dt,$$

with

(A10) $$f_{csub}=\frac{f_{u}+f_{l}}{2},$$

and the time variables being related to their frequency counterparts through the chirp rate:

(A11) $$T_{sub}=\frac{B_{sub}}{\alpha }\equiv \frac{f_{u}-f_{l}}{\alpha },t_{csub}=\frac{f_{csub}}{\alpha }.$$

In this case, it is $f_{c\;sub}$ that is passed to the BP azimuth compression routine as the central frequency. Equation (A9) indicates that a sub-band can be picked through the time-gating of the mixed IF signal. By the same argument as in (A8), the attainable range resolution in this case is

(A12) $$\delta_{rsub}=\frac{c}{2\alpha T_{sub}}=\frac{c}{2B_{sub}},$$

as expected. When we use the entire system band, i.e., $f_{l}=f_{L}$ and $f_{u}=f_{U}$, the expression in (A6) is recovered from (A9) with the help of (A5). Equations (A9)–(A12) are therefore general and applicable to sub-band and full-band processing.

## References

[1] Rodriguez-Morales F., Paden J., Mai H., Occhiogrosso V., Taylor L., Talasila H., Leuschen C., Hale R., Schroeder B., Kolli J. A multi-channel UWB airborne microwave radar for swath mapping of snow layers. Proceedings of the IGARSS 2024–2024 IEEE International Geoscience and Remote Sensing Symposium.

[2] Jutila A., King J., Paden J., Ricker R., Hendricks S., Polashenski C., Helm V., Binder T., Haas C. (2022). High-resolution snow depth on Arctic sea ice from low-altitude airborne microwave radar data. IEEE Trans. Geosci. Remote Sens..

[3] Jenssen R.O.R., Eckerstorfer M., Jacobsen S. (2020). Drone-mounted ultrawideband radar for retrieval of snowpack properties. IEEE Trans. Instrum. Meas..

[4] Kolpuke S., Simpson C.D., Abushakra F., Awasthi A.K., Reyhanigalangashi O., Pierce J., Luong T., Larson J., Taylor D., Braaten D. (2022). Airborne UWB FMCW radar for snow depth measurements. IEEE Trans. Geosci. Remote Sens..

[5] Burr R., Schartel M., Grathwohl A., Mayer W., Walter T., Waldschmidt C. (2021). UAV-borne FMCW InSAR for focusing buried objects. IEEE Geosci. Remote Sens. Lett..

[6] Carin L., Geng N., McClure M., Sichina J., Nguyen L. (1999). Ultra-wide-band synthetic-aperture radar for mine-field detection. IEEE Antennas Propag. Mag..

[7] Hellsten H., Ulander L., Taylor J.D., Taylor J.D. (2000). The CARABAS II VHF synthetic aperture radar. Ultra-Wideband Radar Technology.

[8] VandenBerg N., Sheen D.R., Shackman S., Wiseman D.L. (1996). P-3 ultrawideband SAR: System applications to foliage penetration. Algorithms for Synthetic Aperture Radar Imagery III.

[9] Sletten M.A., Toporkov J.V. (2025). A multi-channel approach to ocean surface current measurement with an ultra-wideband ATI-SAR. IEEE Trans. Geosci. Remote Sens..

[10] Kim S., Pérez V.M., Krieger G., Villano M. Demonstration of frequency-dependent penetration depth estimation using SAR interferometry with wide fractional bandwidth. Proceedings of the IGARSS 2024–2024 IEEE International Geoscience and Remote Sensing Symposium.

[11] Kim S., Krieger G., Villano M. (2024). Volume structure retrieval using drone-based SAR interferometry with wide fractional bandwidth. Remote Sens..

[12] Siqueira P., Tarres M.C., Adam M., Sutherland E., Maloyan J., Seaver T., Tessier R., Tsang L., Borah F., Marshall H.P. SNOWWI: A three-frequency InSAR for snow science applications. Proceedings of the IGARSS 2024–2024 IEEE International Geoscience and Remote Sensing Symposium.

[13] Morrison K., Andre D., Bennett J., Finnis M., Blacknell D., Muff D., Nottingham M., Stevenson C. (2021). A new resonance phenomenon observed in UWB 14–50 GHz SAR and its application to the retrieval of thickness and dielectric properties of scene features. IEEE Trans. Aerosp. Electron. Syst..

[14] Moreira A., Krieger G., Villano M., Younis M., Prats-Iraola P., Zink M. (2025). Spaceborne synthetic aperture radar: Future technologies and mission concepts. Proc. IEEE.

[15] Vehmas R., Dogan O., Ignatenko V., Radius A., Muff D., Leprovost P., Nottingham M., Vilja P., Seilonen T., Arbenina M. A look at spotlight extended dwell—A new level of information content from small-satellite SAR. Proceedings of the IGARSS 2024–2024 IEEE International Geoscience and Remote Sensing Symposium.

[16] Romeiser R., Graber H.C. Advanced ocean wave retrieval from time series of spotlight SAR subaperture images. Proceedings of the EUSAR 2018; 12th European Conference on Synthetic Aperture Radar.

[17] Bhargava A., Sachdeva A., Sharma K., Alsharif M.H., Uthansakul P., Uthansakul M. (2024). Hyperspectral imaging and its applications: A review. Heliyon.

[18] Jezek K.C., Johnson J.T., Tsang L., Brogioni M., Macelloni G., Aksoy M., Kaleschke L., Wang S., Leduc-Leballeur M., Yardim C. (2022). A review of recent developments in low-frequency ultra-wideband microwave radiometry for studies of the cryosphere. Front. Earth Sci..

[19] Johnson J.T., Jezek K.C., Macelloni G., Brogioni M., Tsang L., Dinnat E.P., Walker J.P., Ye N., Misra S., Piepmeier J.R. (2021). Microwave radiometry at frequencies from 500 to 1400 MHz: An emerging technology for Earth observations. IEEE J. Sel. Top. Appl. Earth Obs. Remote Sens..

[20] Skolnik M. (1980). Introduction to Radar Systems.

[21] Cruz H., Véstias M., Monteiro J., Neto H., Duarte R.P. (2022). A review of synthetic-aperture radar image formation algorithms and implementations: A computational perspective. Remote Sens..

[22] Sheen D.R., Johansen E.L., Elenbogen L.P., Kasischke E.S. (1992). The gridded trihedral: A new polarimetric SAR calibration reflector. IEEE Trans. Geosci. Remote Sens..

[23] Wang S., Chen K.S., Sato M. (2020). Performance of SAR polarimetric calibration using hybrid corner reflectors: Numerical simulations and experimental measurements. IEEE J. Sel. Top. Appl. Earth Obs. Remote Sens..

[24] Quegan S. (1994). A unified algorithm for phase and cross-talk calibration of polarimetric data-theory and observations. IEEE Trans. Geosci. Remote Sens..

[25] Levis C.A., Johnson J.T., Teixeira F.L. (2010). Radiowave Propagation-Physics and Applications.

[26] Jackson J.D. (1999). Classical Electrodynamics.

[27] Ament W.S. (1953). Toward a theory of reflection by a rough surface. Proc. IRE.

[28] Vu V.T., Sjogren T.K., Pettersson M.I. (2012). On synthetic aperture radar azimuth and range resolution equations. IEEE Trans. Aerosp. Electron. Syst..

[29] Balanis C.A. (1989). Advanced Engineering Electromagnetics.

[30] Franceschetti G., Lanari R. (1999). Synthetic Aperture Radar Processing.

[31] Stutzman W.L., Thiele G.A. (1998). Antenna Theory and Design.

[32] Frasier S.J., Camps A.J. (2001). Dual-beam interferometry for ocean surface current vector mapping. IEEE Trans. Geosci. Remote Sens..

[33] Toporkov J.V. (2017). A Theoretical study of velocity SAR imaging of a moving, nonstationary scene. IEEE Trans. Geosci. Remote Sens..

[34] Ribalta A. (2011). Time-domain reconstruction algorithms for FMCW-SAR. IEEE Geosci. Remote Sens. Lett..

[35] Galati G., Pavan G., Wasserzier C. (2020). Characterization of back-scattering and multipath in a suburban area after the calibration of an X-Band commercial radar. Sensors.

[36] Long M.W. (2001). Radar Reflectivity of Land and Sea.

[37] Sletten M.A. (1994). Resolution of a phase ambiguity in a calibration procedure for polarimetric radar systems. IEEE Trans. Geosci. Remote Sens..

